# Factors influencing the statistical planning, design, conduct, analysis and reporting of trials in health care: A systematic review

**DOI:** 10.1016/j.conctc.2022.100897

**Published:** 2022-01-29

**Authors:** Marina Zaki, Lydia O'Sullivan, Declan Devane, Ricardo Segurado, Eilish McAuliffe

**Affiliations:** a University College Dublin, Centre for Interdisciplinary Research, Education, and Innovation in Health Systems (IRIS Centre), School of Nursing, Midwifery and Health Systems, University College Dublin, Dublin, Ireland; b Health Research Board – Trials Methodology Research Network (HRB-TMRN), School of Nursing and Midwifery, National University of Ireland, Galway, Galway, Ireland; c School of Medicine and Medical Science, University College Dublin, Dublin, 4, Ireland; d School of Public Health, Physiotherapy and Sport Sciences, University College Dublin, Dublin, 4, Ireland; e School of Nursing and Midwifery, National University of Ireland, Galway, Galway, Ireland; f Evidence Synthesis Ireland, National University of Ireland, Galway, Galway, Ireland

**Keywords:** Systematic review, Trials, Statistics, World Health Organisation, (WHO), Principal Investigators, (PI), Cumulative Index to Nursing and Allied Health Literature, (CINAHL), Joanna Briggs Institute, (JBI)

## Abstract

**Background:**

Trials in health care are prospective human research studies designed to test the effectiveness and safety of health care interventions, such as medications, surgeries, medical devices and other treatment or prevention interventions. Statistics is an important and powerful tool in trials. Inappropriately designed trials and/or inappropriate statistical analysis produce unreliable results and a lack of transparency when reported, with limited clinical use.

**Aim:**

This systematic literature review aimed to identify, describe and synthesise factors contributing to or influencing the statistical planning, design, conduct, analysis and reporting of trials.

**Methods:**

Information sources were retrieved from the following electronic citation databases: PubMed, Web of Science, PsycINFO, and CINAHL and the grey literature repository: OpenGrey. 90 articles and guidelines were included in this review. A narrative, thematic synthesis identified the key factors influencing the statistical planning, design, conduct, analysis and reporting of trials in health care.

**Findings and conclusion:**

We identified three analytical themes within which factors are grouped. These are: “what makes a statistician?“, “the need for dynamic statistical involvement and collaboration throughout a trial – it's not just about the numbers”, “and the “accountability of statisticians in ensuring the safety of trial participants and the integrity of trial data”. While important insights emerged about the qualifications, training, roles, and responsibilities of statisticians and their collaboration with other team members in a trial, further empirical research is warranted to elicit the perceptions of trial team members at the centre of statistics in trials.

## 1 Introduction

Trials are research studies that test the safety and effectiveness of health care interventions. Such interventions include drugs, cells (and other biological products), surgical procedures, medical devices, behavioural treatments, radiological procedures, and interventions for preventative care (WHO, 2020). Findings from trials have the potential to change clinical practice, and the care patients receive. For this reason and to minimise harm to patients, trials must be planned, data collected, and analysed for efficacy and safety, to the highest standard. This is best practice for adherence to sound ethical principles.

The Merriam-Webster Dictionary describes statistics as “*a branch of mathematics dealing with the collection, analysis, interpretation, and presentation of masses of numerical data”* [1]. Statistics, therefore, involves refining the study design to efficiently address the study's research hypothesis while minimising bias, defining data to be collected, appropriately analysing data collected, and interpreting results in such a way as to facilitate clinical decision making. A related and complementary field – trials methodology – looks at how we improve how we plan, do and share the findings of randomised trials [2,3]. Trial methodologists often have a statistics, clinical, epidemiological or scientific background.

The validity of a clinical research study is not judged solely on its results, but also on how the study itself was designed and conducted [4]. Clinical data management (CDM) activities prepare the way for the statistical analysis of a trial. CDM activity in a trial involves the appropriate collection, management, access, and cleaning of clinical research data. Therefore, the integrity of statistical analysis in a trial depends on the quality of the data that is available for analysis [4]; Krishnankutty et al., 2012; Nesbitt, 2004, p.135).

There is also a relationship between statistics and ethics in trials. Ethical issues can affect all stages of a trial. If the statistical considerations of a trial are inadequate, the research will be unethical. This misuse of statistics in the clinical research field may have consequences for trial participants (Altman, 1980) and other resources, including researchers’ time and effort. It is also unethical to publish and disseminate statistical results that may be misleading (Altman, 1980).

Therefore, statistics play an important role in all aspects of a trial. The key team members responsible for the quality of statistical outputs from a trial are statisticians, clinical data managers and principal investigators (PIs). There is a plethora of literature discussing statistical methodologies in trials from a mathematical perspective. Less has been written about the trial team, their communication and collaboration processes, qualifications and training and professional development of statisticians and how these may be influencing the statistical planning, design, conduct, analysis and reporting of trial data. A greater understanding of these critical influencing factors would enable trial sponsors, funders, and those leading trial units and teams to target supports and resources to maximise the trial team's potential (and more particularly the statistician involvement) and the trial environment to improve the efficiency and quality of trials.

## 2 Methods

### 2.1 Review question

What are the factors influencing the statistical planning, design, conduct, analysis and reporting in trials?

## 3 Protocol

The protocol for this systematic review was published in Health Research Board Open Research [5].

### 3.1 Search strategy

We searched published literature in PubMed NCBI, Web of Science, CINAHL and PsycINFO. An information specialist and health librarian liaison were consulted when designing this search strategy. Controlled vocabulary and alternative search terms were used: ‘Subject Headings' in CINAHL, ‘MeSH (Medical Subject Headings)' in PubMed and the ‘Thesaurus of Psychological Index Terms' in PsycINFO. Web of science does not have controlled vocabulary terms. These search terms are reported elsewhere [6].

Primary studies of any study design were included, and studies that collected or analysed primary data through a secondary analysis. Due to the dearth of empirical literature in this field, peer-reviewed discussion, editorial and commentary papers (labelled as ‘text and opinion’) were also included [5].

Papers were included if they discussed one or more of the following factors:

• Roles, responsibilities and tasks of key team members responsible for the statistical elements of the planning design, conduct, analysis and reporting of trials
• Qualifications, training, knowledge, experience and professional development of key team members responsible for the statistical planning, design, conduct, analysis and reporting of trials
• Processes of communication and collaboration between key team members and others, responsible for the statistical planning, design, conduct, analysis and reporting of trials.

If the term ‘trial’ was not present in the full text, the article was excluded at full-text screening. Articles were also excluded if they focused exclusively on novel statistical methodologies, non-human studies, evaluating the effectiveness of an intervention (purely clinical), health economics, regulatory articles (with no reference to factors influencing the statistical elements of a trial) and statistical theory. Protocols and conference abstracts were also excluded. Articles were limited to those published in the English language. Citations and abstracts were downloaded and imported into EndNote (EndNote reference management software, X9.2. Stamford, CT: Thompson Reuters; 2010) and Rayyan [7].

### 3.2 Grey Literature

The OpenGrey (OpenGrey.eu, 2020) platform was used to search for grey literature.

### 3.3 Additional sources

References cited in the included articles were checked to retrieve additional papers that may be relevant (also known as ‘backward citation searching’ (Briscoe, Bethel and Rogers., 2019)). The same screening process described to retrieve relevant articles was applied to articles found in the references of included sources.

#### 3.3.1 Study selection process

**Fig. 1:**
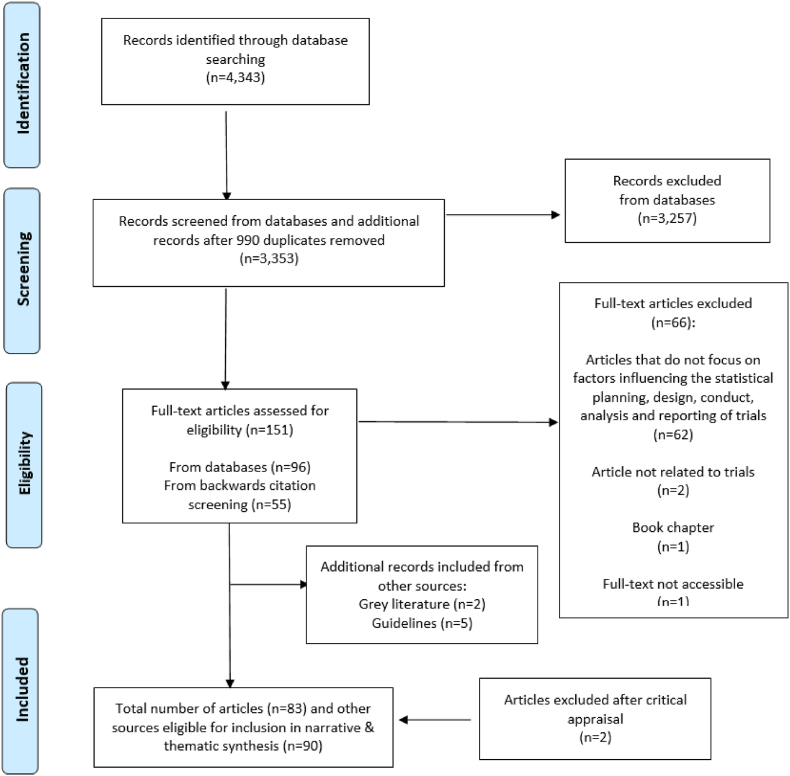
PRISMA flow diagram.

After duplicates were removed (see the Preferred Reporting Items for Systematic Reviews and Meta-Analyses, known as PRISMA, flowchart in Fig. 1), two study team members (MZ and LOS) independently screened the titles and abstracts and reviewed full texts against the eligibility criteria. Consensus meetings were held. In cases of disagreement, a third reviewer (EM) was consulted.

### 3.4 Data extraction

A data extraction template [8] was designed and piloted on three papers. One reviewer (MZ) extracted the data from the included studies. Approximately 10% were reviewed for consistency by a second member of the study team (EM)**.**

### 3.5 Critical appraisal

85 papers selected for data extraction underwent critical appraisal by one reviewer (MZ) prior to inclusion in the data synthesis. A second reviewer (LOS) independently conducted critical appraisal on a random 20% of the articles. A few minor disagreements were discussed in a consensus meeting. Due to the heterogeneity of included articles, five different critical appraisal tools were used to appraise the included articles. The Critical Appraisal Skills Programme (CASP) tool was originally specified in the protocol [5]; however, other checklists were deemed more appropriate after the screening. Appendix A provides a summary of the critical appraisal results (2 articles were excluded at this stage. Therefore, 83 articles were included in this review, see PRISMA). The following essential checklists of appraisal were used:

• Joanna Briggs Institute (JBI) Text and Opinion Critical Appraisal Checklist [9]: 63 articles
• JBI checklist for Analytical Cross-Sectional Studies [10]: 6 articles
• JBI Systematic Review and Research Synthesis checklist [11] (this checklist was used for articles that were not formal systematic reviews but research syntheses style articles): 6 articles
• JBI Critical Appraisal Checklist for Qualitative Research: 3 articles
• Mixed Methods Appraisal Tool (MMAT) [12]: 7 articles

### 3.6 Critical appraisal of text and opinion articles

The majority of text and opinion (discussion) articles developed their arguments logically. It was difficult to assess whether this was conducted analytically, as most articles had no methodology, and therefore it was difficult to discern from where the authors had retrieved their sources. The source of the opinion was identified, and the relevant population's interests were the central focus in all articles. There was a reference to the extant literature in most of the articles, and incongruence with other sources was logically defended. While the JBI Text and Opinion checklist was deemed suitable, a judgement was not made on the item asking whether the article's source was standing in their field of expertise. This was not used as criteria to inform decisions of article inclusion. One article [13] was from a series of articles. Additional information was sought from the other articles in the series. These additional articles did not meet the eligibility criteria but were used as context to understand the background and position of the included article. Two articles [14,15] had no references or citations and were based on expert opinion – these were included.

### 3.7 Data synthesis

The data from quantitative, mixed-methods, research syntheses and text and opinion type articles were synthesised, using a narrative and thematic approach [16,17]. The outcomes from the quantitative articles were not quantitatively synthesised or assessed for heterogeneity.

Articles were grouped into categories based on commonalities in the perspective or context of the articles. Multiple levels of coding [18] were then conducted inductively on the key messages extracted from the articles. Common patterns across articles were identified and directed by the content of the articles [16]. The first level of coding revealed a general sense of the key messages in the articles. In the second level of coding, codes were merged, deleted or renamed to more nuanced and succinct labels [16]. The third level of the analysis process involved the identification of overarching, broader descriptive themes. These descriptive themes were data-driven and remained close to the primary source [19]. In the fourth level, the themes were reviewed [16] and collapsed into more interpretive themes (known as analytical themes) [18]. Once all the findings were synthesised following the thematic analysis process, meanings were then interpreted and presented in the context of the review question. The final column in the data extraction sheet captured notes and reflections during data extraction, and these notes were used to aid the narrative synthesis. Relevant factors were defined as any element impacting, influencing, or contributing to the statistical planning, design, conduct, analysis and reporting in trials. This systematic review was conducted following the PRISMA [20] checklist (see supplementary file).

## 4 Results

### 4.1 Overview of included articles

While some articles specified the context of the study (e.g. Ref. [21] – Swiss teaching hospitals and [22] – Japanese biostatisticians), the majority of articles can be applied to a wide range of contexts. Included articles, after critical appraisal, were: discussion pieces (n = 62), mixed-methods (n = seven), quantitative methods such as surveys and questionnaires (n = five), research syntheses (n = six) and qualitative studies (n = three) (see Appendix B). A narrative synthesis was conducted to address the review question: ‘What factors influence the statistical planning, design, conduct, analysis and reporting of trials?’

The full text of one article could not be located, and an attempt was made to contact the author's department with no response.

Five guidelines from the websites of regulatory authorities and professional associations and a guideline from the Grey Literature were deemed relevant to the scope of this review [5] and were included:

• Food and Drug Administration (FDA)'s 1988: Format and content of the clinical and statistical sections of an application
• Statisticians in the Pharmaceutical Industry (PSI) 1994: Guideline for standard operating procedures (SOPs) for good statistical practice in clinical research
• International Council for Harmonisation of Technical Requirements for Pharmaceuticals for Human Use (ICH) Good Clinical Practice (ICH GCP) 1998: Topic E 9 statistical principles for clinical trials
• International Council for Harmonisation of Technical Requirements for Pharmaceuticals for Human Use (ICH) Good Clinical Practice (ICH GCP) 2015: Topic E 6 (R2) Guideline for Good Clinical Practice
• American Statistical Association (ASA) 2018: Ethical guidelines for statistical practice
• Consolidated Standards of Reporting Trials (CONSORT) 2010: Checklist for reporting randomised controlled trials (RCTs)

**Fig. 2:**
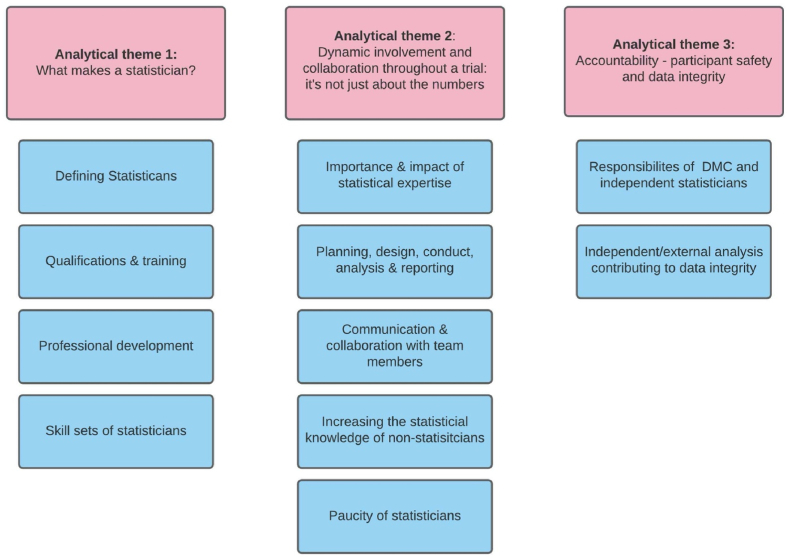
Schematic describing the analytical themes extracted from the literature.

Three analytical themes were extracted from this narrative synthesis. Fig. 2 below provides a schematic of these themes and their sub-themes. Due to the number and length of the findings, some results are reported in Appendix C.

### 4.2 Analytical theme 1: what makes a statistician?

Several factors were identified that contribute to statisticians being appropriately qualified, trained and having the necessary experience and expertise to influence the statistical planning, design, conduct, analysis and reporting of trials. These will be synthesised below.

#### 4.2.1 Defining statisticians

There were differing opinions in the literature about what constitutes a statistician. In its glossary of terms, the ICH E9 guideline defines a “*Trial Statistician*” as “*a statistician who has a combination of education/training and experience sufficient to implement the principles in this guidance and who is responsible for the statistical aspects of the trial.*” (ICH E9, 1998). This definition from ICH E9 contains information about the background of a statistician and is broader than a definition provided by Ref. [23]; where “*Academic Biostatistician*” is defined as*: “Statisticians with a primary appointment with a university or academic health centre, with most clinical trial experience drawn from government* funded *research … "* [23]; p.16).

Important distinctions are made by Ref. [24] between statisticians who perform day to day roles and “*methodological statisticians*”, and by Ref. [25] regarding “*study statisticians*” and *“reporting statisticians”.* [26] report the involvement of *“medical statisticians”* [27], describe *“project statisticians”,* while [28] mentions “*clinical statisticians*” but provide no definition. Statisticians may also be “*co-investigators*” [4,29] or “*consultants”* [30] and such roles and responsibilities should be outlined [29].

#### 4.2.2 Qualifications and training

The literature was ambiguous as to the qualifications and experience required to become a statistician. While the US, UK and Canada had defined degree programmes in statistics with clear pathways of the roles and skillsets of statisticians [31], no “*European-wide understanding*” was established [31]. Morgan and colleagues in the European Federation of Statisticians in the Pharmaceutical Industry (EFSPI) discuss their efforts to define a “*qualified medical statistician”* as someone with a university qualification with appropriate statistical content (or equivalent) and having adequate experience in medical statistics [31]. The concern, however, is the variation of educational systems across Europe and how “*statistical content*” should be defined in the context of full-time equivalent years of statistics [31]. Therefore, a person's degree didn't need to be in statistics but one where there was sufficient “*statistical content*” [31]. On the other hand, statisticians on Data Monitoring Committees (DMCs) in the USA, are required to have education and experience in biostatistics, statistics or a relative discipline at a master's level or higher, study (trial) statisticians are required to have an “*advanced degree in statistics or biostatistics*” and “*medical statistician*” needed a degree in statistics or equivalent (e.g. mathematics or a related subject) with a minimum of three years experience in medical statistics” [32]. While Morgan and colleagues did not explicitly state the level of the degree [33], describe their study, where the majority of statisticians had a PhD or equivalent, while only a few did not have a masters level [33].

Statisticians sitting on Research Ethics Committees (RECs) (committees that review ethics applications before trials begin) in the UK had been advised to consider the requirements of the Chartered Statisticians qualifications (Williamson et al., 2020). Such qualification requirements described by Williamson provide more detail than the above descriptions by Refs. [31,32] - where statisticians serving on RECs were to have an undergraduate degree with over 50% statistical courses and 5 years post-degree experience, or a master's degree in statistics with 4 years post-degree experience, of which 3 of those 4 or 5 years should be at a moderately senior level [26]. Specific forms of additional training for biostatisticians were described, for example, in adaptive designs or trial simulations [34,35], for their roles on RECs [26,36] and in condition specific research e.g., ageing research [37]. The ICH GCP E9 guideline was found to be an important source of statistical guidance in regulated trials internationally and important material for the training of statisticians in the pharmaceutical industry [22,38]. Further details on the statistical queries reviewed by statisticians on RECs and the ethical duties of statisticians allowing for their professional integrity (Ethical Guidelines for Statistical Practice, 2018) are described in Appendix C.

#### 4.2.3 Professional development

Following the qualifications and training of statisticians, came the importance of ensuring the professional development of statisticians in the field. The Statisticians in the Pharmaceutical Industry (PSI) is a professional organisation in the UK that aims to promote professional standards of statistical issues in the pharmaceutical industry. From 1990 to 1991, and revised again in 1994, a Professional Standards Working Party created a set of Standard Operating Procedures (SOPs). Their objectives were to: ensure statisticians in the pharmaceutical industry are aware of Good Statistical Practice (GSP) principles, to promote adherence to these principles in the application of statistics to trials, to publicise such principles for the benefit of other clinical research professionals, to ensure compliance with Good Clinical Practice (GCP) and to provide guidance in the preparation of SOPs to meet regulatory requirements when it comes to collecting, processing, analysing and reporting of trial data (Good Statistical Practice in Clinical Research: Guideline Standard Operating Procedures, 1994). While no authors explicitly referred to these standards and principles from PSI, the potential for statisticians to “*uphold a professional Code of Practice*”, “*Code of Conduct or a Code of Ethics*” was described [39]; Pyke et al., 2010). The above principles are reported to contribute to recognising the professional duty of statisticians in terms of their “*value, quality and integrity*” [39]; Pyke et al., 2010) when working in trials.

#### 4.2.4 Skill sets of statisticians

The literature describes skillsets statisticians are required to have, including good communication skills [31,[39], [40], [41]], have good *“customer service, management, and interpersonal skills”*, consultancy skills, collaborative, negotiation, entrepreneurial, written and oral skills, deliver their tasks on time, adhere to allocated budgets, have problem-solving skills, and can be responsive and be “*creative, innovative and flexible”* [39,[42], [43], [44]]. While statisticians are called to have leadership skills [39,45,46], one author [27] describes how the project statistician interacts with the trial leadership, but unlike the other authors, does not refer to the statistician themselves becoming a leader. Statisticians exercising appropriate judgement in trials is described by Ref. [47] and in ICH E9 (1998) and are called to have experience in implementing guidelines [39].

### 4.3 Analytical theme 2: dynamic involvement and collaboration throughout a trial: it's not just about the numbers

A recurring factor reported to influence the quality of a trial positively was that statisticians should be involved from the beginning and in every stage through to the reporting [25,28,30,33,42,[47], [48], [49], [50]]; Meeks et al., 2018). Recognising the importance of statistical expertise and early collaboration between statisticians, investigators and the clinical data management team was a key finding identified across several articles [26,40]; Crewson and Applegate., 2001 [29,35,[51], [52], [53], [54]]; Meeks et al., 2018; [55].

#### 4.3.1 Importance and impact of statistical expertise

Statistical concepts in trials methodology has gained increased understanding amongst physicians [30]. This can, in part, be attributed to training and awareness of non-statisticians, in, for example, medical schools [53] and requirements for publishing [40]. Statisticians were once seen in a “*service*” capacity [43], seen as “*compilers of data*” [42]. In contrast, now they are “*accepted as contributing colleagues*” [40] and “*important contributors and drivers of innovation*” [43]. The expectations and scope of statisticians' tasks have changed significantly [43], particularly given the evolving nature of complex statistical methodology [33,53]. Despite many researchers recognising the value of statisticians, however, one concern identified was the unwillingness of some researchers to involve statisticians, where 61% of researchers believed they had the necessary skills within their research team and therefore did not need a statistician or a methodologist [33]. Likewise [56], note how there may not be a need for a statistician in every research study. Therefore, it is important to improve the educational efforts of non-statisticians.

#### 4.3.2 Planning, design, conduct, analysis and reporting of trials

The need for statistician involvement was reported in the planning stage of writing funding and grant applications and justifying budgets [4,29,35]. Despite statistics being critical to research proposals [49], some authors raised the concern of insufficient funds for statisticians [4,34,57,58], where their salaries were questioned - some (37% of the sample studied) reported not being paid for their work [33].

Statisticians also play a critical role in creating clear and comprehensive statistical analysis plans (SAPs) [59].

Several empirical studies have demonstrated the value of statistician involvement in the reporting of trials. This includes ensuring that trials are reported honestly and accurately and that ‘negative’ results are published [60,61]. The involvement of statisticians were strong predictor for study publications being correctly labelled as randomised controlled trials (RCTs) [62], had fewer “*scientific difficulties*” in the design, data management and analysis and publication writing [63] and found that the presence of a statistician allowed for more appropriate analytical methods sections of publications [64]. On the other hand, papers with no statisticians or epidemiologists were more likely to be rejected by journals compared to papers that did have methodological input (71% vs 57% respectively), before being sent for peer review [33] or have statistical inaccuracies in the design and analysis sections of publications [53].

Some authors (Matcham et al.*,* 2010 [34,55,62,65]; discuss the benefits of the CONSORT (Consolidated Standards of Reporting Trials) guidelines when reporting trials, also to be used in conjunction with statistical guidelines [66].

However, a number of articles [33,67,68]; Pyke et al., 2010) discussed the disadvantages often faced by statisticians regarding the lack of acknowledgement of statistician's work in publications.

#### 4.3.3 Communication and collaboration of statisticians with team members

The positive influence of communication and collaboration in the context of this review question was two-fold: statisticians benefited from learning about the clinical elements, and clinicians benefited from learning about the statistical aspects [4]. The motivation of the clinical research team (trial statisticians, clinicians, patient representatives, peer advocate groups and regulators [34,69] when collaborating (ICH E9, 1998), which otherwise could “*jeopardise progress*” [70] is a consistent theme throughout the literature identified in this review.

The role of the statistician in quality assurance and quality control is further described in a guideline by the Statisticians in the Pharmaceutical Industry (PSI) (Good Statistical Practice in Clinical Research: Guideline Standard Operating Procedures, 1994) and by Refs. [47,57].

A number of authors [46,60,71,72] discuss the positive impact of statisticians explaining statistical principles to non-statisticians. The lack of standardised terminology was a disadvantage in the development of trials [35,73].

Several authors mentioned time constraints [34,36,54,57,65,73]. The need for realistic estimates of statisticians' efforts to complete tasks were explained [29,34]. Statisticians may have limited time to create innovative methods in statistical theory and trials methodology [57], to correct the formatting of a study's database [54] and to support or address concerns from team members about the methodology and logistical consequences of adaptive designs [65,73].

#### 4.3.4 Increasing the statistical knowledge of non-statisticians

These findings are included in Appendix C.

#### 4.3.5 Paucity of statisticians

A prevalent concern and factor influencing the statistics of trials, however, was the difficulty accessing statisticians and the shortage of statisticians in RECs and DMCs [33,36,54,58,65,[74], [75], [76]], which is a negative factor influencing the implementation of adaptive designs in trials, for example [65]. There was incongruence in the literature, where a shortage of statisticians in publicly-funded trials in the USA and UK was reported [65,74,77] but the availability of statisticians was found to have improved in Hong Kong [48]. Pocock further describes the potential for company-sponsored trials to have a number of statisticians involved in a trial, but how limitations in the funding of publicly funded trials inhibit the separation of such roles [74].

There was a dearth of statisticians in regulatory agencies in Europe reported by a number of authors [42,75] and in the Ministry of Health and Welfare in Japan [22]. This is in contrast to Ref. [31] on behalf of the European Federation of Statisticians in the Pharmaceutical Industry (EFSPI), who state that regulatory authorities in Europe are employing additional statisticians to allow for the statistical review of marketing authorisation applications.

### 4.4 Analytical theme 3: accountability of statisticians in ensuring the safety of trial participants and the integrity of their data

Several authors [25,27,66,78]; Matcham et al., 2010 [32,77,[79], [80], [81], [82]]; (b)) outline the active roles of statisticians in DMCs or data safety monitoring boards (DSMBs). A number of factors were identified as contributing to the integrity of a trial, from a statistical perspective, including: the roles and responsibilities of statisticians in DMCs, roles, benefits and concerns of independent statisticians and how this influences the interim analysis of a trial and external analysis of data contributing to integrity.

The 2001 FDA draft guidance, entitled: ‘Guidance for Clinical Trial Sponsors on the Establishment and Operation of Clinical Trial DMCs’, poses a specific model for the operation of the DMC [83]. In this model, study statisticians or statisticians involved in trial leadership are separated (independent/external) from statisticians that conduct interim analyses to report to DMCs [25,83,84]. This independence ensures the DMC can operate appropriately to protect the safety of the trial participants, without threatening the integrity of the trial, and therefore relies on the integrity of statisticians [15,25,58]. It is often the case that the unblinded analyses of interim data are only available to the DMC members; otherwise, potential changes to the protocol could be influenced by knowledge of the interim data, and the involvement of the statistician in this decision-making process could raise suspicions about whether such decisions are biased [79,83].

#### 4.4.1 Responsibilities of DMC and independent statisticians

The DMC statistician, along with other members of the DMC, evaluates interim reports and make decisions about whether there is sufficient evidence to recommend the trial be stopped early or modified [74]. This DMC statistician (and their colleagues on the committee) should have no other involvement while the trial is ongoing [14,74]. Further details about the responsibilities of a DMC statistician are provided by Ref. [32].

On the other hand, the independent statistician conducts the interim analyses of a trial and prepares the interim report for the DMC [74]. The independent statistician is usually the only person able to link the treatment code to the trial database [74] but should not be involved in decisions about modifications made to the trial and ideally, should not be an employee of the sponsor to alleviate bias [14]. Despite the large congruence in the literature that independent statisticians should conduct interim analyses, there are reports of limited resources and a lack of experienced statisticians to serve on DMCs [66,77,85], due to the associated costs [14]. Some authors, therefore, advocated for the interim analysis to be conducted by: statisticians part of the sponsor company as mentioned [74,86], by the trial statistician and the PI [66] or by the group statistician [58] but play no role in making changes to the trial design [86] or to trial management aspects [58].

Therefore, amongst the biggest concerns identified was the extent to which statisticians conducting interim analysis and reporting to DMCs should be independent of trial investigators and sponsors (Matcham et al., 2010; [79,86].

One concern raised by a number of authors [25,27,74,83,85,86] is that statisticians are independent to the study and independent of the company, with no prior involvement, may not be as familiar with, for example, the therapeutic area of interest or the design elements of the trial (e.g. the protocol [81] and the database [25]), as the sponsor statistician would. These independent statisticians could fail to understand or interpret the data sufficiently or may not recognise potentially important results that are worthy of follow-up or exploratory analyses to protect the safety of the trial participants, resulting in a possible loss of information provided to the DMC during the interim analysis [58,83].

[25] describes how a ‘firewall’ separates the reporting statistician from the investigators, once the trial begins [25] or between the trial statistician on the steering committee and the statistician and other personnel working for the sponsor who will perform the interim analyses for the DMC [78]. Firewalls ensure that no one other than the intended personnel has access to interim data [83].Such firewalls are enforced through transparent documentation, operating guidelines, and a signed confidentiality statement [27]. However, Siegel et al. noted concerns about compliance with these firewall standard operating procedures (SOPs) [14].

#### 4.4.2 Independent/external analysis contributing to data integrity

Discussion on the independence of those involved in the data analysis was not limited to DMCs however. The Journal of the American Medical Association (JAMA) author instructions state that at least one author (independent of any commercial funder) should indicate that they have had access to the data and can take responsibility for the accuracy of the data analysis, and therefore the integrity of the data [15]; Pyke et al., 2010). The reason for this statement from JAMA was due to several “*high-profile trials*” that had clear issues with data integrity, and therefore also warranted independent statistical analysis by an academic biostatistician for industry-sponsored trials (Pyke et al., 2010; [44]. In 2013 however, JAMA reverted this policy as they found no significant changes in study results and therefore, additional, independent statistical analyses was not required [44]. An argument however is made that independent analyses of data “*creates a strong incentive for honest behaviour*” and “*raises the quality of data analysis and interpretation*” (Pyke et al., 2010). It was reported to be the responsibility of the investigator to question whether their study's original data and statistical report will be reviewed by an independent statistician [87]. Where there is no capacity for an independent statistician, the investigator can conduct the analysis [13]. Further findings related to conflicts of interest and the importance of statistician integrity when not disclosing the results of interim analyses are described in Appendix C.

## 5 Discussion

The main recommendation extracted from most articles was the need for consistent and active involvement of statisticians throughout a trial, not only at the analysis stage. Several other important recommendations on improving the statistical elements of trials emerged. These are illustrated in Table 1 below.

**Table 1:** Recommendations.

Recommendations for best practice	Authors
Statisticians should be involved in all trial stages, from the beginning (i.e. conceptualisation and planning) to end (i.e. reporting and dissemination).	[25,28,30,33,42,[47], [48], [49], [50]]; Meeks et al., 2018
Statisticians who sit on RECs should have appropriate training.	[26,36]
More general, formal training should be made available for statisticians to advance their statistical methodology knowledge	[31,38,39,44]
Statisticians should be involved in writing budgets and trial funding grant applications.	[4,29,35]
Statisticians should be involved in preparing and designing case reports forms and interacting with data management team members and database developers.	Crewson and Applegate, 2001 [4,24,42,49,51,72];
Statisticians should create clear and comprehensive statistical analysis plans (SAPs), adhere to them while analysing a trial and pre-publish them to reduce bias.	[59]; Snow et al., 2015, Pyke et al., 2010 [59],
Statisticians should be involved in reporting trials and ensure honest and accurate reporting of all trial results (regardless of the outcome being positive or negative) and be transparent in the methods sections of manuscripts.	[[60], [61], [62], [63]]. [33,53,64]
Statisticians should actively communicate with fellow statisticians and other team members (including clinicians) by understanding the others' perspective	[30,53,71]
Statisticians should avoid the use of technical statistical jargon and ensure team members understand the statistical aspects.	[4,33,42,46,71]
Non-statisticians involved in trials should have an understanding of the fundamentals of research methods and statistical reasoning.	[4,21,41,47,48,55,65,70,88]. [45]. [66,80]
Study statisticians or statisticians involved in trial leadership should be independent or external to statisticians conducting interim analyses and reporting to DMCs. A firewall should separate the sponsor/steering committee/the primary trial statistician and the DMC or independent statistician.	[25,27,83,84]

### 5.1 Paucity of statisticians and insufficient funding for them, despite recognition of their importance

The paucity of statisticians working in clinical research and the resulting impact on the statistics of a trial was described as a significant concern [33,58,74,75]; Dimairo, [76]; Boote et al., 2015; [54], although improvements in other contexts were noted [48]. For example [42,75], describe the insufficient numbers of statisticians in regulatory authorities investigating trial applications while [31] report additional statisticians in the same setting. A more nuanced understanding for the reasons behind a shortage of statisticians in DMCs, RECs [26,36]; Atici and Erdemir., 2007) and in a trial itself is not discussed in an in-depth manner in these articles, and a deeper exploration may be beneficial. The implications of not including statisticians in research studies were also found to affect the quality of a trial [62], as manuscripts with no statistician were more likely to be rejected by journals [33] and have statistical inaccuracies [53]. This may be because statisticians are more familiar with methodological aspects than their peers. Statisticians' time constraints were a key concern [34,36,54,57,65,73], and it was acknowledged that statisticians had tremendous workloads.

Recommendations are for statisticians to contribute to funding and grant applications [4,29]. This encouraged their involvement in the planning stages of a research study, and ensured their work is justified in the budget. While authors recognised how biostatistics and biostatisticians are critical to research study proposals [24,49], the concern of insufficient funding was a key factor that hinders statistician involvement and stifles statistical innovation in trials [34,57,58]. Three authors [4,26,33] questioned the salaries of statisticians. Future research is recommended to unpack the reasons of insufficient funding, which are not apparent from this review.

### 5.2 Statistical collaboration

It can be inferred from this narrative synthesis that collaboration in trials significantly improves the statistical planning, design, conduct, analysis and reporting of trials. The evolving role of the statisticians and their increasing acceptance amongst colleagues [30,40,45] was a recurring theme in this review. This was attributed to the mandatory requirements for their involvement (ICH E9, 1998 [49]; and the increasing recognition of their value in driving innovation and positively influencing research studies [43]. Such views rebut the old fashioned perception of statisticians being “*compilers of data*” [42] or as merely providing a “*service*” [43]. This willingness to engage statisticians and include them as authors in publications was not noted by all authors [26,33]. This incongruence in the literature could be explored further. A single author, [56]; advocates for improving the statistical education of non-statisticians thus reducing the need to include a biostatistician in every research study. However, this opinion was in contrast the findings of this review and further discussion on the types of studies claimed by Scales and colleagues to not need a statistician is merited. While [39] alludes to how such negotiation skills are key to influencing collaboration in trials, the authors do not describe explicitly how other skills affect a trial.

### 5.3 Independence and DSMBs

The responsibilities of statisticians in DMCs (or DSMBs) was prevalent in this review [25,27,58,66,78]; Matcham et al., 2010 [32,77,79,80,82]; (b)). The main area of debate identified is that statisticians who conduct the interim analyses and report to DMCs should be independent of the sponsor/company (Matcham et al., 2010 [79]; with other authors presenting contradictory views that ‘independent statisticians’ can be employees of a company but have no say in the design of a trial [74,86]. While there were benefits to ensuring that this statistician is as independent (or external) to the sponsor/company as possible, concerns were raised that they might lack sufficient knowledge about the therapeutic area of interest, the design elements of the trial and would fail to understand the data appropriately to protect the safety of trial participants [25,27,81,83,85,86]. Similarly, authors expressed the implications of subtle and non-subtle forms of communication between statisticians with knowledge of interim data and other colleagues involved in trial management [25,74,77]. Recommendations of putting up a ‘firewall’ to separate statisticians with knowledge of interim data from other members of the team [25,84] was nonetheless met with concerns by other authors [14] who questioned the compliance with firewall SOPs. However, the risk of bias through revealing interim results is reduced with firewalls and independent statisticians, but the accountability falls on the statisticians to maintain their independence.

### 5.4 Limitations

The authors acknowledge the high volume of articles retrieved from backwards citation screening. These articles may have been missed from the original databases, potentially due to missed controlled vocabulary terms. Forward citation screening was not conducted, as it was not deemed feasible within the review timeframe. No restrictions were placed on the years of articles retrieved from the databases, so some of the recommendations from articles may have now become common practice. While the authors attempted to differentiate between information that was specifically trials related and others that were regarding broader medical or clinical research, this was not always explicit. This review only included articles published in English, so it is possible that articles with relevant information were excluded.

### 5.5 Future work

As most articles identified by this review are text and opinion, more empirical research is warranted in this field to address the gaps identified. For example, it would be interesting to explore: the influence (if any) of COIs on the statistical elements of a trial, the obstacles behind employing additional statisticians in trials, and the barriers to effective statistical training of non-statisticians. Since disparity in statistician training was an important theme in this review, consideration should be given as to whether there should be a minimum standard regarding training programmes for trial statisticians. Given how critical statisticians are to the success of clinical trials, there is a strong rationale for statistician training programmes to be publicly funded. The distinction between publicly and industry-funded trials was not extensively referred to in the literature identified in this systematic review, so this would be an interesting area for future empirical research.

## 6 Conclusions

This systematic review retrieved articles and guidelines that explored factors influencing the statistical planning, design, conduct, analysis and reporting of trials. Through conducting a narrative, thematic synthesis, three analytical themes were identified. These themes are entitled: “what makes a statistician?“, “the need for dynamic statistical involvement and collaboration throughout a trial: it's not just about the numbers” and “the accountability of statisticians in ensuring the safety of trial participants and maintaining the integrity of their data”. Given the supporting context and resources, the review demonstrates a high degree of consensus about how statistics can and should contribute to the planning, design, conduct, analysis, and reporting of trials. Based on these, a set of recommendations has been set out in Table 1. Further empirical research in this field is needed to understand where trial practice falls short of these recommendations and how trial sponsors, funders and leaders of trials units and teams might begin to bridge these gaps.

## Funding

This work was supported by the Health Research Board – Trials Methodology Research Network (HRB-TMRN), Ireland [grant number: TMRN-2014-1] and the School of Nursing, Midwifery and Health Systems, University College Dublin, Ireland. The funding source had no involvement in the study design, collection, analysis, and interpretation of data in the report's writing or in the decision to submit the article for publication.

## CRediT author statement

**Marina Zaki:** Conceptualisation, Methodology, Formal analysis, Investigation, Data Curation, Writing- Original draft preparation, Writing- Review and Editing, Visualisation, Project Administration. **Lydia O'Sullivan:** Methodology, Formal analysis, Investigation, Data Curation, Writing- Original draft preparation, Writing- Review and Editing, Visualisation. **Declan Devane:** Conceptualisation, Methodology, Writing- Original draft preparation, Writing- Review and Editing, Supervision, Funding Acquisition. **Ricardo Segurado:** Writing- Review and Editing, Supervision. **Eilish McAuliffe:** Conceptualisation, Methodology, Investigation, Writing- Original draft preparation, Writing- Review and Editing, Visualisation, Supervision, Project Administration, Funding Acquisition.

## Declaration of interests

☒ The authors declare that they have no known competing financial interests or personal relationships that could have appeared to influence the work reported in this paper.

☐The authors declare the following financial interests/personal relationships which may be considered as potential competing interests:
