# Supplementary material for: Factors influencing the statistical planning, design, conduct, analysis and reporting of trials in health care: A systematic review
Source: Contemp Clin Trials Commun. 2022 Jan 29;26:100897. doi: 10.1016/j.conctc.2022.100897 (PMC8842005; doi:10.1016/j.conctc.2022.100897)
Supplement: Multimedia component 1 [file mmc1.docx]

Tables for systematic review manuscript

**JBI checklist for analytical cross-sectional studies**

|  | **Clearly defined criteria for inclusion in sample?** | **Study subjects and setting described in detail?** | **Exposure measured in a valid and reliable way?** | **Objective, standard criteria used for measurement?** | **Confounding factors identified?** | **Strategies to deal with confounding factors stated?** | **Outcomes measured in a valid and reliable way?** | **Appropriate statistical analysis used?** | **Decision** |
| --- | --- | --- | --- | --- | --- | --- | --- | --- | --- |
| **Altman, Goodman and Shroter., 2002** | Yes | Yes | N/A | N/A | Yes | Yes | Yes | Yes | Include |
| **Baldi et al., 2018** | Yes | Yes | N/A | N/A | No | N/A | Yes | Unclear | Exclude |
| **Cullati et al., 2016** | Yes | Yes | N/A | N/A | Yes | Yes | Yes | Yes | Include |
| **Dimairo, Julious et al., 2015** | Yes | Yes | N/A | Yes | N/A | N/A | Yes | Yes | Include |
| **Perneger et al., 2004** | Yes | Yes | Yes | Yes | N/A | N/A | Yes | Yes | Include |
| **Tidwell et al., 2019** | Yes | Yes | Yes | N/A | N/A | N/A | Yes | Yes | Include |

**JBI Qualitative research checklist**

|  | **Congruity between stated philosophical perspective and research methodology?** | **Congruity between research methodology and the research question or objectives?** | **Congruity between research methodology and the methods used to collect data?** | **Congruity between the research methodology and the representation and analysis of data?** | **Congruity between the research methodology and the interpretation of results?** | **Statement locating the researcher culturally and theoretically?** | **Influence of the researcher on the research, and vice-versa addressed?** | **Participants and their voices adequately represented?** | **Research ethical according to current criteria or, for recent studies, and evidence of ethical approval by an appropriate body?** | **Conclusion drawn in the research report flow from the analysis, or interpretation, of the data?** | **Decision** |
| --- | --- | --- | --- | --- | --- | --- | --- | --- | --- | --- | --- |
| **Dimairo, Boote et al., 2015** | Yes | Yes | Yes | Yes | Yes | Unclear | Unclear | Yes | Yes | Yes | Include |
| **Guetterman et al., 2015** | Unclear | Yes | Yes | Yes | Yes | No | No | Yes | Unclear | Yes | Include |
| **Harman et al., 2015** | Unclear | Yes | Yes | Yes | Yes | Unclear | Unclear | Yes | N/A | Yes | Include |

**JBI Text and Opinion checklist**

|  | **Source of the opinion clearly identified?** | **Source of opinion have standing in the field of expertise?** | **Interests of the relevant population the central focus of the opinion?** | **Stated position the result of an analytical process, and is there logic in the opinion expressed?** | **Reference to the extant literature?** | **Incongruence with the literature/sources logically defended?** | **Decision** |
| --- | --- | --- | --- | --- | --- | --- | --- |
| **Adams-Huet and Ahn., 2009** | Yes | Not assessed | Yes | Yes | Yes | Yes | Include |
| **Archdeacon et al., 2014** | Yes | Not assessed | Yes | Yes | Yes | Yes | Include |
| **Atici and Erdemir., 2007** | Yes | Not assessed | Yes | Yes | Yes | Yes | Include |
| **Boulesteix et al., 2017** | Yes | Not assessed | Yes | Unclear | Yes | Yes | Include |
| **Bierer et al., 2016** | Yes | Not assessed | Yes | Yes | Yes | Yes | Include |
| **Bradstreet et al., 1992** | Yes | Not assessed | Yes | Yes | Yes | Yes | Include |
| **Breslow, 1978** | Yes | Not assessed | Yes | Yes | Yes | Yes | Include |
| **Bryant, 2004** | Yes | Not assessed | Yes | Yes | Yes | Yes | Include |
| **Califf, 2004** | Yes | Not assessed | Yes | Unclear | Yes | Yes | Include |
| **Califf, 2016** | Yes | Not assessed | Yes | Yes | Yes | Yes | Include |
| **Calis et al., 2017** | Yes | Not assessed | Yes | Yes | Yes | Yes | Include |
| **Carroll, 2009** | Yes | Not assessed | Yes | Yes | Yes | Unclear | Include |
| **Cirrincione et al., 2014** | Yes | Not assessed | Yes | Yes | Yes | Yes | Include |
| **Coffey et al., 2012** | Yes | Not assessed | Yes | Yes | Yes | Yes | Include |
| **Crewson and Applegate., 2001** | Yes | Not assessed | Yes | Yes | Yes | Yes | Include |
| **DeMets and Fleming, 2004** | Yes | Not assessed | Yes | Yes | Yes | Yes | Include |
| **DeMets et al., 2004** | Yes | Not assessed | Yes | Yes | Yes | yes | Include |
| **Dhar and Kianifard, 2006** | Yes | Not assessed | Yes | Yes | Yes | Yes | Include |
| **Dixon et al., 2011** | Yes | Not assessed | Yes | Yes | Yes | Yes | Include |
| **Ellenberg., 1990** | Yes | Not assessed | Yes | Yes | Yes | Yes | Include |
| **Ellenberg., 2012** | Yes | Not assessed | Yes | Yes | Yes | Yes | Include |
| **Ellenberg and George., 2004** | Yes | Not assessed | Yes | Yes | Yes | Yes | Include |
| **Fleming et al., 2017** | Yes | Not assessed | Yes | Yes | Yes | Yes | Include |
| **Gordon, 2008** | Yes | Not assessed | Yes | Yes | Yes | No | Include |
| **Grieve., 2002** | Yes | Not assessed | Yes | Yes | Yes | Yes | Include |
| **Grobler et al., 2001** | Yes | Not assessed | Yes | Yes | Yes | Yes | Include |
| **Hattemer-Apostel, 2008** | Yes | Not assessed | Yes | Yes | Yes | Yes | Include |
| **Hughes et al., 2008** | Yes | Not assessed | Yes | Yes | Yes | Yes | Include |
| **Ioannidis et al., 2014** | Yes | Not assessed | Yes | Yes | Yes | Yes | Include |
| **James, 1980** | Yes | Not assessed | Yes | Yes | Yes | Yes | Include |
| **Juluru, 2015** | Yes | Not assessed | Yes | Yes | Yes | Yes | Include |
| **Lewis, 2008** | Yes | Not assessed | Yes | Yes | Yes | Yes | Include |
| **Li et al., 2018** | Yes | Not assessed | Yes | Yes | Yes | Yes | Include |
| **Lin and Lu., 2014** | Yes | Not assessed | Yes | Yes | Yes | No | Include |
| **Maurer, 2005** | Yes | Not assessed | Yes | Yes | Yes | Yes | Include |
| **Manamley et al., 2016** | Yes | Not assessed | Yes | Yes | Yes | Yes | Include |
| **Matcham et al., 2010** | Yes | Not assessed | Yes | Yes | Yes | Yes | Include |
| **Meeks et al., 2018** | Yes | Not assessed | Yes | Yes | Yes | No | Include |
| **Morgan (EFSPI) et al., 1999** | Yes | Not assessed | Yes | Yes | Yes | Yes | Include |
| **Munro, 1993** | Yes | Not assessed | Yes | Yes | Yes | Unclear | Include |
| **Pallman et al., 2018** | Yes | Not assessed | Yes | Yes | Yes | Yes | Include |
| **Phillips et al., 2013** | Yes | Not assessed | Yes | Yes | Yes | Yes | Include |
| **Pocock, 2004** | Yes | Not assessed | Yes | Yes | No | No | Include |
| **Powers and Fleming., 2009** | Yes | Not assessed | Yes | Yes | Yes | Yes | Include |
| **Pyke et al., 2010** | Yes | Not assessed | Yes | Yes | Yes | Yes | Include |
| **Rockhold, 2006** | Yes | Not assessed | Yes | Yes | No | No | Include |
| **Sato and Yoshimura., 1998** | Yes | Not assessed | Yes | Yes | Yes | Yes | Include |
| **Senn and Julious., 2009** | Yes | Not assessed | Yes | Yes | Yes | Yes | Include |
| **Sherrill et al., 2009** | Yes | Not assessed | Yes | Yes | Yes | Yes | Include |
| **Siegel et al., 2004** | Yes | Not assessed | Yes | Yes | No | No | Include |
| **Sismondo, 2009** | Yes | Not assessed | Yes | Unclear | Yes | Yes | Include |
| **Snapinn et al., 2004** | Yes | Not assessed | Yes | Yes | Yes | Yes | Include |
| **Thall, 2002** | Yes | Not assessed | Yes | Yes | Yes | Yes | Include |
| **Todd et al., 2020** | Yes | Not assessed | Yes | Yes | Yes | Yes | Include |
| **Tsang, 1998** | Yes | Not assessed | Yes | Yes | Yes | Yes | Include |
| **Tyson et al., 2016** | Yes | Not assessed | Yes | Yes | Yes | Yes | Include |
| **Vail., 1998** | Yes | Not assessed | Yes | Yes | Yes | Yes | Include |
| **Van Ness et al., 2010** | Yes | Not assessed | Yes | Yes | Yes | Yes | Include |
| **Welzing et al., 2007** | Yes | Not assessed | Yes | Yes | Yes | Yes | Include |
| **Whatley-Smith et al., 2014** | Yes | Not assessed | Yes | Yes | Yes | Yes | Include |
| **Williamson et al., 2000** | Yes | Not assessed | Yes | Yes | Yes | Yes | Include |
| **Wittes., 2004** | Yes | Not assessed | Yes | Yes | Unclear | Unclear | Include |
| **Zelen, 2006** | Yes | Not assessed | Yes | Yes | Yes | Yes | Include |

**JBI Systematic reviews and research syntheses checklist**

|  | **Review question clearly and explicitly stated?** | **Inclusion criteria appropriate for the review question?** | **Search strategy appropriate?** | **Sources and resources used to search for studies adequate?** | **Criteria for appraising studies appropriate?** | **Critical appraisal conducted by two or more reviewer’s independently?** | **Methods to minimise errors in data extraction?** | **Methods used to combine studies appropriate?** | **Likelihood of publication bias assessed?** | **Recommendations for policy and/or practice supported by the reported data?** | **Specific directives for new research appropriate?** | **Decision** |
| --- | --- | --- | --- | --- | --- | --- | --- | --- | --- | --- | --- | --- |
| **Delgado-Rodriguez et al., 2001** | Yes | Yes | Yes | Yes | Yes | No | No | Yes | N/A | Yes | Yes | Include |
| **Koletsi et al., 2012** | Yes | Yes | Yes | Yes | Yes | Yes | Yes | Yes | N/A | Yes | Yes | Include |
| **Kloukos et al., 2015** | Unclear | Yes | Yes | Yes | Yes | Yes | Yes | Yes | N/A | Yes | N/A | Include |
| **Papageorgiou et al., 2019** | Yes | Yes | Yes | Yes | Yes | Unclear | Yes | Yes | N/A | Yes | Yes | Include |
| **Scales et al., 2005** | Yes | Yes | Yes | Yes | Yes | Yes | Yes | Yes | Yes | Yes | Yes | Include |
| **Sosa et al., 2009** | Yes | Yes | Yes | Yes | Yes | Yes | Yes | Yes | N/A | Yes | Yes | Include |

**MMAT checklist**

|  |  |  | **4. Quantitative descriptive** | | | | |  |
| --- | --- | --- | --- | --- | --- | --- | --- | --- |
|  | **S1. Clear research questions?** | **S2. Collected data allow to address the research questions ?** | **4.1 Sampling strategy relevant to address the research question?** | **4.2 Sample representative of the target population?** | **4.3 Appropriate measurements?** | **4.4 Low risk of non-bias response?** | **4.5 Appropriate statistical analysis to answer the research question?** | **Decision** |
| **Crowley et al., 2018** | Yes | Yes | Yes | Yes | Yes | Yes | Yes | Include |
| **Snow et al., 2014** | Yes | Yes | Yes | Yes | Yes | Can’t tell | Yes | Include |
| **5. Mixed methods** | | | | | | | | |
|  | **S1. Clear research questions?** | **S2. Collected data allow to address the research questions ?** | **5.1. Adequate rationale for using a mixed methods design to address the research question?** | **5.2. Different components of the study effectively integrated to answer the research question?** | **5.3 Outputs of the integration of qualitative and quantitative components adequately interpreted?** | **5.4. Divergences and inconsistencies between quantitative and qualitative results adequately addressed?** | **5.5. Different components of the study adhere to the quality criteria of each tradition of the methods involved?** | **Decision** |
| **Calis et al., 2017 (b)** | Yes | Yes | Yes | Yes | Yes | Yes | Yes | Include |
| **Jaki et al., 2013** | Yes | Yes | No | Can’t tell | Can’t tell | No | Can’t tell | Exclude |
| **Gamble et al., 2017** | Yes | Yes | Yes | Yes | Yes | Yes | Yes | Include |
| **Mawocha et al., 2017** | Yes | Yes | Yes | Yes | Yes | Can’t tell | Yes | Include |
| **Meurer et al., 2016** | Yes | Yes | Yes | Yes | Yes | Yes | Yes | Include |
