# Supplementary material for: Factors influencing the statistical planning, design, conduct, analysis and reporting of trials in health care: A systematic review
Source: Contemp Clin Trials Commun. 2022 Jan 29;26:100897. doi: 10.1016/j.conctc.2022.100897 (PMC8842005; doi:10.1016/j.conctc.2022.100897)
Supplement: Multimedia component 2 [file mmc2.docx]

**Synopsis table of included articles (n=84: 83 articles plus 1 source from grey literature)**

| **Author(s),  Year** | **Aim/ Objective/ Research Question** | **Perspectives/ Setting/**  **Location** | **Evidence Type** | **Methodology** | **Synopsis of Findings and Recommendations** |
| --- | --- | --- | --- | --- | --- |
| Adams-Huet and Ahn  2009 | “Discuss topics and situations that clinical investigators and statisticians commonly encounter while planning a research study and writing the statistical methods section”. | Statisticians and clinical investigators | Text and opinion | None reported. | Early collaboration between clinical investigators and statisticians is crucial when preparing research funding applications with a solid statistical methodology. |
| Altman, Goodman and  Schroter  2002 | ‘Investigation of the nature and frequency of statistician involvement in medical research and its relation to the final editorial decision’. | Statistical expertise in medical research | Empirical | A short questionnaire administered to authors of research articles submitted to BMJ and Annals of Internal Medicine from May to August 2001: asked if they received statistical assistance, the nature of these contributions, and the reasons if no statistical input was obtained. | While statistical input to medical research is strongly recommended, it is inconsistently obtained. Statisticians are often not involved until the data analysis stage and may not be acknowledged in the authorship of manuscripts. |
| Atici and Erdemir  2007 | ‘For research to be evaluated using biostatistics intensively from ethical and scientific points of view, a biostatistics expert is necessary on RECs. Developments in Turkey are used as examples.’ | Biostatisticians in RECs | Text and opinion | None reported. | RECs need a biostatistics expert to evaluate research from ethical, scientific and statistical perspectives. |
| Archdeacon et al.  2014 | “Devised recommendations to help sponsors optimise their premarket safety systems to reduce the number of uninformative expedited reports and ensure recognition of important safety issues for an investigational drug as early as possible in development”. | FDA, industry sponsors and safety reporting | Text and opinion | Internet-based survey of industry representatives, 2-day expert meeting to discuss survey results. Developed recommendations with FDA working group | Set of recommendations for patient safety monitoring during drug and biologic development programmes. A working group of biostatisticians explored methodological issues regarding analyses, handling masked data and expedited reporting of serious adverse events. |
| Boulesteix, Wilson and Hapfelmeier  2017 | “Draw an analogy between clinical trials and real-data-based benchmarking experiments in methodological statistical science, with data sets playing the role of patients and methods playing the role of medical interventions”. | Trials and data-based benchmarking studies | Text and opinion - Debate | None reported. | Analogy between clinical trials and real-data-based benchmarking experiments discussed. The role of the statistician in the design and interpretation of studies explored e.g. preparing inclusion criteria and assessing bias. |
| Bierer et al.  2016 | ‘This article provides a practical guide to sponsors, institutions, and individuals  responsible for, or serving on, a DMC.’ | DMC | Text and opinion | 24 key professionals from academia and not-for-profit and commercial organizations that included investigators,  statisticians, patient advocates, and ethicists met in 2013 to define the essential elements of planning, coordinating, and populating  a DMC. | Operational and practical considerations of DMCs described. Qualifications of DMC statisticians, sponsor (trial) statisticians and the statistical reporting team explored, along with their roles and responsibilities during charter development, in inaugural and ad-hoc DMC meetings. |
| Bradstreet  1992 | "The objective of our course is to provide a general understanding of statistical  reasoning, an appreciation of the statistician's role in drug development, and to facilitate effective communication between statisticians and clinical researchers" | Statistics appreciation course for clinical researchers in Merck, Sharpe and Dohme | Text and opinion | Merck Clinical Biostatistics Training Group in November, 1988, developed a statistics training course for clinical researchers to understand the statistician’s role. | Increased communication and cooperation between departments and less duplication of work in Merck after an interactive statistical course for clinical researchers. |
| Breslow  1978 | “The intent is to present a certain perspective on the statistician’s role, with the idea that communication between clinician and statistician may be improved if each has a fuller understanding of the other's viewpoint" | Statistician’s role in cooperative clinical research | Text and opinion | None reported. | Multifaceted role of the statistician in cooperative clinical research described, including: sample size calculation, experimental design, stopping rules, data processing and analysis. Results in more rigorous protocols, improvements in data quality and comprehensive analysis of results. Medical researchers have learnt these tasks with the help of software. |
| Bryant  2004 | ‘In this commentary, it is argued that the benefits of such a policy (draft FDA guidance) would be minimal in the cooperative Group setting and are far outweighed by the tangible benefits of including the study statistician in the  monitoring process.’ | Trial statistician in DMC – US Cancer Cooperative Group setting | Text and opinion | None reported. | Statisticians who prepare and present interim analyses to DMCs should be independent of the trial sponsor. Not involving the study statistician in the monitoring process can negatively impact the quality of the study monitoring and may affect the validity of results. |
| Califf  2016 | “Pragmatic clinical trials: Emerging challenges and new roles for statisticians” | Statisticians in pragmatic trials | Text and opinion | None reported. | Biostatisticians should be involved in reviewing research portfolios, incorporating ‘quality by design’ principles and mining of aggregate knowledge. Must nurture the biostatistical workforce, create a deeper understanding of clinical context amongst statisticians, facilitate collaboration, communication, training and education to enhance capacity. |
| Califf et al.  2004 | “Investigator Participation in Clinical Research” | Investigator’s in clinical research | Text and opinion | None reported. | Discussion of the role of investigator in designing and reporting results of a trial, guided by the SAP, and potential conflicts of interest. An independent statistician is preferrable. |
| Calis et al.  2017 (a) | “The objectives of the DMC project were: to clarify the purpose of DMCs and the rationale for their use, develop best practice recommendations for the operation and optimal conduct of independent DMCs, describe effective communication practices between independent DMCs and trial stakeholders and identify strategies for preparing next-generation DMC members” | Data monitoring committees in clinical trials | Discussion on empirical approach | Survey of DMC members, focus groups, 2-day expert meeting | Recommendations addressed the use, conduct, communication practices, preparation and training of members, including biostatisticians and the involvement of statistical data analysis centres in DMCs. |
| Calis et al.  2017 (b) | ‘Clinical Trials Transformation Initiative conducted a survey to assess the current use and conduct of DMCs, as well as the training practices for DMC members. In addition, key focus groups were convened to gain an in-depth understanding of the needs and best practices related to DMC use in the modern context’ | DMCs | Empirical – mixed-methods | A total of 143 respondents (DMC members, sponsors involved with the organisation of DMCs and statistical data analysis centre representatives) completed the online survey. Focus groups were conducted on 42 participants including: DMC members; patients and/or patient advocate DMC members;  IRB and US FDA representatives; industry, government, and non-profit  sponsors; and statistical data analysis centre representatives. | DMCs should be an independent advisory body, representing the interests of trial participants and must have access to unblinded data. No standards or guidelines as to qualifications of DMC members exist and very little to none formal training. Necessary experience of biostatistician on DMCs described. |
| Carroll  2009 | “This paper highlights some of the important features relating to outcome trial sample sizing and making a number of simple recommendations aimed at ensuring a better, common understanding of the interplay between sample size and power and the final result required to provide a statistically positive and clinically persuasive outcome” | Statisticians and sample sizes in outcome trials | Text and opinion | None reported. | The different roles of the statistician in advising the trial team are described, including the design, conduct, analysis and reporting of a trial. In drug development, the importance of the statistician in ensuring medical and other non-medical statistical colleagues appreciate the sample sizing of the trial thus reducing the risk of a statistically significant yet clinically weak result. |
| Cirrincione, Smith and Pang  2014 | ‘To review study design issues related to clinical trials led by oncology nurses, with special attention to those conducted within the cooperative group setting; to emphasise the importance of the statistician’s role in the process of clinical trials” | Methodological considerations in clinical trials | Text and opinion | Retrieved data sources from ‘studies available at clinicaltrials.gov using experimental designs that have been published in peer-reviewed journals’. | Implications for nursing clinical trials described including: early collaboration and continued communication amongst study team and the statistician is key to develop and conduct appropriately designed studies. Important for: objectives, design, data capture, accrual monitoring, data accuracy, analysis, interpretation and reporting of results. |
| Coffey et al.  2012 | "To provide a brief overview of adaptive designs (ADs), describe the rationale behind conducting the workshop and summarise the main recommendations that were produced as a result of this workshop" | Statisticians and trial researchers in adaptive trials - USA | Text and opinion | Workshop with representatives from the NIH, FDA, EMA, pharmaceutical industry, non-profit foundations, patient advocacy community and academia. Recommendations provided. | Logistical barriers of adaptive designs in trials identified, including the need for education and improved infrastructure and the resulting impact on the DSMB. Role of a biostatistician is described. |
| Crewson and Applegate  2001 | ‘This paper introduces the basic principles essential for a successful data collection effort.’ | Data collection in radiology research | Text and opinion | None reported | The importance of coordination of tasks, preparation and consistent implementation of the data collection process in studies is described, and the resulting improvements in efficiency. |
| Crowley et al.  2018 | “Examined how early career investigators prior and desired training aligns with recently funded cardiovascular outcomes research" | Early career investigators training in cardiovascular research | Empirical – mixed methods | Literature review, survey to understand training, grant applications examined. | Discussion on the desire of early-career investigators in cardiovascular outcomes research to pursue biostatistical training. |
| Cullati et al.  2016 | "the objective of this study was to assess the self-reported frequency of difficulties encountered by medical researchers while conducting research and to identify factors associated with their occurrence" | Patient enrolment in clinical research in Switzerland | Empirical – cross-sectional survey | A self-administered online questionnaire among principal investigators (PIs) of 996 protocols approved by the research ethics committee (REC) in Geneva. Difficulties across the research process were rated. | Report of difficulties which investigators face most frequently when conducting research, including data analysis and interpretation. The presence of a statistician and their impact is described. |
| Delgado-Rodriguez et al.  2001 | “This study assessed several methodological aspects related to the quality of published controlled clinical trials (CCTs) in relation to the participation of an epidemiologist/biostatistician”. | Epidemiologists/ biostatisticians | Research synthesis | 467 CCTs from four medical leading journals (NEJM, Lancet, JAMA, BMJ) from 1993–1995 were identified by handsearching. The speciality of authors were obtained from their affiliation. | Findings suggest that the presence of an epidemiologist/biostatistician improves the quality (at least of the reports) of clinical trials. |
| DeMets and Fleming 2004 | “address the motivation for having the preparation and presentation of interim analyses be conducted by an independent statistician not a member of the DMC and who is not the trial's lead or steering committee statistician" | Statisticians in DMCs | Text and opinion | None reported. | The motivation for preparation and presentation of interim analyses of a trial carried out by an statistician who is independent of the DM and the trial’s lead and steering committees. |
| DeMets et al.  2004 | “This paper is based on a workshop sponsored by Duke Clinical Research Institute (January 17-18,2003) to discuss several controversial issues that have arisen recently, primarily in response to the FDA draft guidance document.” | DMC members | Text and opinion | None reported. | DMC draft guidelines by FDA raise issues including the role of the independent statistician, which are discussed. |
| Dhar and Kianifard  2006 | To describe the format and content of a course to teach statistics to clinical research staff in a pharmaceutical company | Clinical research staff in a pharmaceutical company | Text and opinion | Statistics course consisting of three 3-hour modules, 2-4 weeks apart, for non-statisticians included: basic statistical concepts, statistical hypothesis testing and sample size computation. | The benefits of a statistical training course with non-statisticians in a pharmaceutical company are discussed, including facilitating communication when designing trials and interpreting results. |
| Dimairo, Julious et al.  2015 | ‘This study explored barriers, concerns, and potential facilitators to the appropriate use of Adaptive Designs (ADs) in confirmatory trials among key stakeholders.’ | Adaptive designs in trials | Empirical – survey | Three cross-sectional, online parallel surveys between November 2014 and January 2015 conducted. Surveys based on findings from in-depth interviews of key research stakeholders, predominantly in the  UK, and targeted Clinical Trials Units (CTUs), public funders, and private sector organisations. | Obstacles hindering the use of adaptive designs in confirmatory trials include: experience in adaptive designs, lack of practical implementation knowledge and applied training. |
| Dimairo, Boote et al. 2015 | ‘In this paper, we explore key stakeholders’ experiences, perceptions and views on barriers and facilitators to the use of ADs in publicly funded confirmatory trials.’ | Adaptive designs in trials | Empirical - qualitative | Semi-structured interviews of 27 key stakeholders including: CTU directors, funding  board and panel members, statisticians, regulators, chief investigators, data monitoring committee members and  health economists) conducted. | Perceived barriers to the use of adaptive designs in publicly funded confirmatory trials include: lack of knowledge and experience, lack of applied training, employment contracts, lack of understanding of adaptive designs and lack of statistical expertise. |
| Dixon et al.  2011 | "The purpose of this article is to describe the considerations that motivated the development of the new policy, summarise current DSMB policies and ongoing harmonisation efforts across the 4 divisions and offer some recommendations for DSMB operations” | DSMB members in allergy and infectious  disease clinical trials | Text and opinion | A working group reviewed DBMB responsibilities, policies and operations from 2005 to 2009. This paper provides an analysis and summary of the final pol­icy document from this working group, which described current DSMB activities. | DSMB membership must include an experienced statistician. Role of DSMB in early stopping and oversight of access to interim analysis results described. |
| Ellenberg  1990 | ‘We comment on: (1) the acceptance by the medical community that biostatistical concepts are an integral part of sound medical research; (2) the sometimes unrealistic expectations placed on biostatistics and biostatisticians given limited resources and/or limited control; (3) some controversies among biostatisticians; and (4) the need for emphasizing the design and implementation phases of medical investigations.’ | Biostatisticians in medical research | Text and opinion | None reported. | Role and relationships (collaboration) between biostatistician and clinician/investigators described to ensure appropriate design and analysis in the research proposal. Importance of teaching and having experience are described. |
| Ellenberg and George  2004 | "We describe these potential conflicts, and the advantages and disadvantages of approaches that might be taken to minimize them. We have invited commentary on this issue from several statisticians with substantial experience in clinical trials and interim data monitoring." | Statisticians in DMCs | Text and opinion | None reported. | Possible conflicts of interest for statistician performing the interim analyses and presenting it to DMC described and approaches to reduce these. |
| Ellenberg  2012 | Protecting clinical trial participants and protecting data integrity: are we meeting  the challenges? | Data integrity in trials | Text and opinion | None reported. | Separate (‘independent’) statistician analysing interim data and reporting to the DMC is a controversial topic but allows for the protection of data integrity. |
| Fleming et al.  2017 | "This position paper, offers recommendations to improve the DMC process” | DMC experts in clinical trials | Text and opinion | Expert panel from academia, industry and government sponsors, and regulatory agencies discussed best practices and operating principles for effective functioning of DMCs. | Prospective DMC members need better training. Discussion on confidentiality of interim data, access to unblinded efficacy and safety data. Knowledge and experience of independent statistical group discussed to generate timely reports. |
| Gamble et al.  2017 | "To develop recommendations for a minimum set of items that should be addressed in SAPs for clinical trials, developed with input from statisticians, previous guideline authors, journal editors, regulators and funders" | SAPs in clinical trials | Mixed-methods study.  Commentary article | Literature search to identify guidance on SAPs, survey of current practice across UK Clinical Research Collaboration-registered trial units (n=46), Delphi survey (n=73: statisticians, guideline authors, journal editors and regulators) to establish consensus on SAP. Consensus meeting with expert panel members. Piloting of SAP guidance in 5 trials. | ICH E9 - contribution of statistician to design and analysis of trials is essential. Review and piloting of SAP checklist to ensure fit for purpose and appropriate to the needs of statisticians who author and implement SAPs. Value of SAPs discussed. |
| Gordon  2008 | “In this paper, we describe several variants of a training module in ICH E9 for new statisticians, combining directed reading with a game-based exercise, which have proved to  be highly effective and enjoyable for course participants” | Training of statisticians | Text and opinion | Format of training module: directed readings, group discussions, exercise-based training and games. Class size up to 12 and larger groups also. | New statisticians joining the pharmaceutical industry require a thorough understanding of the ICH E9 guideline. Lecture-style, traditional training methods are not effective. Exercise-based training described: directed reading with game-based exercise provided to be effective and enjoyable. |
| Hattemer-Apostel  2008 | "This article sets out to shed light on the interfaces between the core areas monitoring, data management and statistics, and illustrates the hidden potential for improvement" | Data management, statistics and monitoring in industry | Text and opinion | None reported. | Discrepancies in trial data to be clarified before evaluation by a biostatistician and interpretation by medical experts. Role of statistician described: aggregates data from CRF for safety and efficacy; prepares SAP, uses validated programmes and software and ensures statistical principles appropriately applied to trials. Statisticians should have adequate training and experience. Statistical methods often not understood by non-statisticians. Collaboration between data manager and statistician in data acquisition, source data verification and data processing, preparation of CRF and database structure. |
| Guetterman et al.  2015 | ‘The purpose of this qualitative study was to explore the perspectives and experiences of stakeholders as they reflected back about the interactive ADAPT-IT adaptive design development process, and to understand their perspectives regarding lessons learned about the design of the trials and trial development’ | Adaptive designs in trials | Empirical – qualitative | Semi-structured interviews were conducted with 10 key stakeholders and observations of the process – from June to August 2013. | Education of the research community is required to understand adaptive design methodology and ensure its understanding when planning trials. |
| Grieve  2002 | "In this paper, I look at barriers which prevent statisticians contributing as much as they should and look at potential solutions" | Statisticians in the pharmaceutical industry | Text and opinion | None reported. | ICH E6 and ICH E9 guidelines requires appropriately qualified and experienced statisticians throughout the trial process. Need to involve statisticians in the design and methodology, not just in the analysis. The lack of statisticians within the regulatory agencies Europe discussed. |
| Grobler et al.  2001 | "This article discusses the role  of the statistician in the data management process" | Statisticians in data management | Text and opinion | None reported. | Statisticians can ensure a protocol is written in a way that enhances data management. Input of statistician into design of CRF can allow for collection of appropriate and complete data. Statistician can contribute to the process of quality control of the database. |
| Harman  2015 | “We aimed to explore the roles and responsibilities of the Trial Steering Committee (TSC) from the perspective and experience of an expert panel of clinicians, trial methodologists and statisticians, and to use this to inform revision of the Medical Research Council (MRC) TSC terms of reference.” | Trial steering committees | Empirical | Expert panel (n = 7) comprising of statisticians, clinicians and trial methodologists with  prior TSC experience. Two full-day meetings. Discussions were summarised. | Discussion of the implications of the same statistician being involved in writing the DMC report (having knowledge of unblinded data) and being on the TSC. HTA (Health Technology Assessment) and MRC guidance discussed. Paucity of suitably experienced statisticians in the UK. |
| Hughes  2008 | "We would like to draw attention to what we believe is a major deficiency in European regulatory agencies that are responsible for reviewing applications to market new medicines across the whole of the European Union." | Statisticians in European regulatory agencies | Text and opinion | None reported. | All regulatory agencies who undertake review of applications should employ full time statisticians to enable appropriate  licensing and labelling decisions. |
| Ioannidis et al.  2014 | “We propose potential solutions for these problems, including improvements in protocols and documentation, consideration of evidence from studies in progress, standardisation of research efforts, optimisation and training of an experienced and non-conflicted scientific workforce, and reconsideration of scientific reward systems.” | Researchers in biomedical and public health research | Text and opinion | None reported. | Involvement of investigators in statistical elements of trial discussed, including the concern that statisticians and methodologists are only involved occasionally, which often results in flawed designs and analyses. Investigators have little formal training in statistics. |
| James  1980 | “This paper describes a system for the review, operation, and conduct of multicenter clinical research in a wide variety of medical specialty areas. In particular, it stresses the importance of core biostatistical coordinating  centers where biostatisticians and other key support personnel constitute an  integral part of the planning and development, implementation, conduct, and reporting of many cooperative studies in diverse disciplines. The paper also describes the interaction of various review bodies and a check and balance system to promote sound management and opportunity for the exchange of clinical and biostatistical methodology in the cooperative study setting.” | Clinical trials in the veteran administration | Text and opinion | None reported. | Role of biostatisticians described: identifying experimental design, define hypothesis, determine endpoints and variables to be collected, specify inclusion/exclusion criteria, compute sample size, develop randomisation process and project methods to analyse, report and monitor data as it is collected. Define operational procedures of data collection, data flow, quality control processes and final analysis plus willingness and dedication of investigators to the study. Representation of biostatistician discussed. |
| Juluru  2015 | “In this review, we present some practical guidelines and best practices for preparing data that can reduce the work of subsequent analysis.” | Data collection in clinical research | Text and opinion | None reported. | Advanced analyses in clinical research studies often requires a statistician. Difficulty accessing statistician for non-analyses tasks described. Importance of early consultation and roles of statistician in sample size calculation, deciding research question, and creating best practices for data collection and preparation. |
| Koletsi et al.  2012 | ‘In this study, we aimed to investigate whether studies published in orthodontic journals and titled as randomized clinical trials are truly randomized clinical trials. A second objective was to explore the association of journal type and other publication characteristics on correct classification’ | RCTs in dentistry journals | Empirical | 6 journals were hand searched for clinical trials labelled in the title as randomized from 1979 to July 2011. | 112 trials were included - 64% of those with statistician involvement were correctly labelled as RCTs and only 21% had no statistician involvement. |
| Kloukos et al.  2015 | “The purpose of this study was to examine the reporting quality of randomised controlled trials  (RCTs) published in prosthodontic and implantology  journals.” | RCTs in prosthodontics and implantology | Research synthesis | 30 issues of 9 journals were searched for RCTs, from 2005–2012. The reporting quality was assessed using a modified CONSORT statement checklist. | Out of 147 RCTs – a statistician/methodologist was involved in 37.4% of RCTs. The involvement of a statistician/methodologist were significantly associated with the CONSORT scores. |
| Lewis  2008 | “In this article we examine the progress in the professional development of statisticians working in the pharmaceutical sector over the past decade.” | Professional development of statisticians in the pharmaceutical industry | Text and opinion | None reported. | ICH E9 has had a positive impact on the professional development of statisticians – opportunity to debate statistical issues, built trust and respect of statisticians. The necessary education, training, experience and skills of statisticians summarised. Responsibility of trial statisticians to collaborate with other trial professionals and ensure statistical principles are applied appropriately discussed. Formal training courses, coaching, experiential learning and keeping up to date with advances in core statistical methodology described. Practical and ethical considerations as well as legal requirements and SOPs in a company noted. |
| Li et al.  2018 | "In this article, we discuss topics that young clinical researchers and statisticians commonly encounter" | Clinical researchers and statisticians | Text and opinion | None reported. | Statisticians role in: choosing primary outcomes, formulating primary study hypotheses, determining clinically significant differences, proposing study design, calculating sample sizes, performing power calculations, writing SAPs, carrying out statistical analyses, interpreting results, safeguarding integrity of the study when protocol is amended, developing CRF and database specifications. Methods of finding a statistician described. Grant applications for large RCTs require research teams to include biostatisticians before obtaining grant funding. Teamwork between early clinical researcher and statistician important, can also increase opportunities to begin a clinical research career. Ethical principles in the context of roles also discussed. |
| Lin and Lu  2014 | “This report describes the role, formation and operation of DMC.” | Biostatisticians in DMC | Text and opinion | None reported. | IRB often lack the data management and statistical support to assess the safety risks associated with trials. Role of the statistician in the interim analyses described – ideally a statistician independent of the trial management team and sponsor. |
| Manamley et al.  2016 | “This article is one of a series of articles developed by the EFSPI (European Federation of Statisticians in thePharmaceutical Industry) and PSI (Statisticians in the Pharmaceutical Industry) Data Sharing Working Group. The Working Group consists of medical research statisticians from the pharmaceutical and biotechnology industries and academia, with the intention of providing knowledge and insights regarding the practical challenges and opportunities of accessing research data for re-analysis or secondary research purposes.” | Statisticians and data sharing | Text and opinion | None reported. | Crucial role of statisticians in data sharing and design of study discussed, including their skills with regard to data transparency. Independent statistical analysis discussed as well as role of statistician in ensuring data privacy. |
| Mawocha et al.  2017 | “In this research, we sought to characterize the perspectives of key stakeholders during the development process of confirmatory-phase adaptive clinical trials within an emergency clinical trials network and to build a model to guide future development of adaptive clinical trials.” | Adaptive trials in emergency research: USA  Adaptive Designs Advancing  Promising Treatments into Trials (ADAPT-IT) project | Empirical – qualitative | Between 2011-2013: an ethnographic, qualitative approach to evaluate key stakeholders’ views about the adaptive clinical  trial development process. | Roles of collaboration and communication described in the context of adaptive clinical trials. |
| Matcham et al.  2010 | “In this paper we set out what we consider to be a set of best practices for statisticians in the reporting of pharmaceutical industry-sponsored clinical trials.” | Statisticians and reporting in industry trials | Text and opinion | None reported. | Discussion on the role of statisticians in the reporting of trial including: author responsibilities and recognition, publication timing, conflicts of interest; freedom to act; full author access to data; trial registration and independent review. |
| Maurer  2005 | “The aim of this paper is to show that even in a highly regulated area such as clinical research and development in pharmaceutical industry, there are needs and ample opportunities for statisticians and other medical informatics professionals to further creatively develop and implement methods in order to support the collection, analysis and interpretation of clinical data.” | Statistics in the pharmaceutical industry | Text and opinion | None reported. | Statisticians only marginally involved in planning stage, analysing and reporting trials to supporting the interpretation of results, implementation of novel methodology. Statisticians in the industry improve planning and decision-making process chiefly by introducing, adapting, and developing the ideas and principles of 'design of experiments' to clinical development. Interdisciplinary collaboration (statisticians and clinicians) discussed. Role of methodological statisticians in novel approaches described. |
| Meurer et al.  2016 | “We sought to understand perceptions about understanding, acceptability, and scientific validity of adaptive clinical trials (ACTs).” | Adaptive trials in USA | Empirical – mixed methods | January – August 2011: convergent mixed methods design using survey and mini-focus group data collection  procedures to elucidate attitudes and opinions among “trial community” stakeholders. | FDA most likely and clinicians least likely to understand adaptive designs. Better understanding may not mean acceptance of ACTs. Clinicians and statisticians felt that reviewers in NIH may not be trained or familiar with adaptive design methods. Huge range of experience among statisticians and limited coverage of adaptive designs and clinical trial simulation within most PhD biostatistics curricula. Critical implication for biostatisticians involves communication among themselves and to clinical colleagues. |
| Meeks et al.  2018 | “In this review, we focus on translational medicine (TM) studies in the context of the National Cancer Institute’s National Clinical Trials Network trials and offer a description of the genesis of TM components, methods in sample acquisition and biomarker research, and a guide to funding mechanisms, in order to provide a blueprint for future TM research protocols.” | Translational medicine in urology cancer | Text and opinion | None reported. | Investigators, similar to statisticians, should be involved in the design process. Close collaboration with a cooperative biostatistical core is vital to a successful TM project - ensure sample size of trial can result in an answerable research question. This collaboration is also important in the development of a TM protocol. |
| Morgan – EFSPI  1999 | “"It is hoped that this outline definition will give guidance to companies, to regulatory authorities and to individual statisticians in terms of providing statistical support to trial and other pharmaceutical development activities and that it may provide a foundation for future development of the statistical profession within the pharmaceutical industry"” | Statisticians in the pharmaceutical industry | Text and opinion | None reported. | Developing role of statisticians in pharmaceutical industry has resulted in the employment of more statisticians. Mention of ICH GCP guidelines necessitating qualified, experienced statisticians. No European-wide understanding of this definition for statistician - different educational systems. Skills and qualifications required are explained. |
| Munro  1993 | “We also need to address the problems of reviewers who need access to information that may not justify publication in journals for a general readership. At present, publication bias by researchers, sponsors, and editors limits their knowledge of and access to relevant information.” | Publishing findings in clinical research | Text and opinion | None reported. | ABPI guidelines do not suggest investigator has responsibility to publish results. Declaration of Helsinki and Royal College of Physicians note the responsibility of the investigator to ensure that there is prior agreement with any financial sponsor that they will not seek to influence the publication of the research results. Mention of whether original data and statistical report will be inspected by an independent statistician. |
| Pallman et al.  2018 | “This tutorial paper provides guidance on key aspects of adaptive designs that are relevant to clinical triallists.” | Statisticians in adaptive trials | Text and opinion | None reported. | Barriers to implementing adaptive designs in trials include a lack of expertise or experience of statisticians. Discussion on involving a statistician with experience of Ads and blinding of those with a vested interest. Investigators should be aware of the design’s implications and potential downfalls in interpreting and reporting the findings correctly. |
| Papageorgiou et al.  2019 | “The aim of this study was to explore the methods, reporting and transparency of clinical trials in orthodontics and compare them to the field of periodontics, as a standard within dentistry.” | Clinical trials in dentistry | Empirical - Cross-sectional bibliographic study | 150 orthodontic and 150 periodontics trials published in 2017-2018 were assessed and statistically analysed to explore the conduct and reporting of these trials. | Statistician should be involved in the research team from a very early stage to help with: calculation sample size, the SAP, and the trial protocol. Trials that had a statistician involved (22.3%) reported less statistically significant results. |
| Perneger et al.  2004 | “We assessed methodological skills of medical researchers and identified factors associated with higher skill levels.” | Medical researchers in Switzerland | Empirical | Cross-sectional mail survey of 409 participants at two Swiss teaching hospitals who self-assessed their capability to carry out 26 research tasks. | Clinical researchers in Geneva and Lausanne described moderate levels of research skills,  particularly in the field of statistical analysis. Doctors who conduct research do not need to have all methodological skills but should have a team that does. |
| Phillips et al.  2013 | “Multiplicity: discussion points from the Statisticians in the Pharmaceutical Industry (PSI) multiplicity expert group” | Statisticians in the pharmaceutical industry | Text and opinion | May 2012: Committee of Health and Medicinal Products issued a concept paper on the need to review points for documenting multiplicity issues in trials. PSI held a one-day expert group meeting in January 2013. | Statisticians need to clearly explain the rationale behind aspects such as multiplicity to researchers with limited statistical expertise and describe this in the protocol. Statisticians play a key role in interacting with regulators. |
| Powers and Fleming 2009 | “We discuss issues that  investigators should address in trial design, conduct, and analysis  to evaluate overall effects or effects within subgroups according to  causative organisms.” | Clinical trials in infectious diseases | Text and opinion | None reported. | Role of investigators in designing and conducting trials to minimise bias and plan for analysis e.g. subgroup analyses and ensuring trial integrity described. Investigators should design a trial with an adequate sample size in each subgroup, appropriate chose analysis population and address likelihood of increased false positive results due to multiple comparisons. |
| Pocock  2004 | “A major trial needs three statisticians:  why, how and who?” | Statisticians in trials | Text and opinion | None reported. | Statistical and scientific integrity of a clinical trial is improved by having: study statistician, DMC statistician and independent statistician. Their roles and importance of communication between them are discussed. |
| Prescott et al.  1999 (from Grey Literature) | “To assemble and classify a comprehensive bibliography of factors limiting the quality, number and progress of RCTs. To collate and report the findings, identifying areas where firm conclusions can be drawn, and identifying areas where further research is required.” | UK. Health Technology Assessment | Report | A systematic review, covering the period 1986–1996, looked at  the diversity of factors  limiting the quality, number and progress of RCTs. | Lack of experienced statisticians means it is possible for all trials to have DMCs. Role of the statistician in conducting the interim analysis discussed. There is a need for statistical training for trialists. Statistical refereeing should be valued career wise in both industry and academia. |
| Pyke et al.  2010 | “We examine the precursors to this Editorial, as well as its immediate and lasting effects for statisticians, for the manner in which statistical analysis is carried out, and for the industry more generally” | Statisticians reporting in industry trials | Text and opinion | None reported. | Discussion on roles of independent non-industry statisticians re-analysing industry studies, minimising bias, importance of transparency, pre-specification of analysis plans, authorship of statisticians and conflicts of interest. Potential for statisticians to uphold a professional code of practice. |
| Rockhold  2006 | “Requiring ‘independent’ statistical  analyses for industry sponsored trials?” | Independent statisticians in industry trials | Text and opinion | None reported. | Discussion on JAMA policy in 2004 – data analysis to be conducted by statisticians at an academic centre rather than only by statisticians employed by the sponsor (pharmaceutical company). |
| Sato and Yoshimura 1998 | “This paper briefly introduces the current Japanese statistical guideline, and identifies its problems” | Biostatisticians working in the pharmaceutical industry in Japan | Text and opinion | None reported. | Japanese guideline : “Guideline for the statistical analysis in clinical trials (1992) discussed. Statisticians in Japan do not have sufficient training of experience to conduct statistical tasks without this guideline. Statistician’s role from design to reporting of the trial and in collaboration, ensuring results are presented correctly and upholding ethical principles described. No full-time biostatisticians in the Japanese Ministry of Health and Welfare. |
| Scales et al.  2005 | “We provide a systematic assessment of the quality and accuracy of statistical reporting in the urology literature.” | Clinical researchers, statistical methods in the urology literature | Systematic assessment of the literature | All research publications with adult humans in 4 leading urology journals were reviewed in their August 2004 issues. Standardised evaluation form developed. Two independent, blinded reviewers. 97 articles included. | Lack of statistical and research knowledge amongst authors, lack of involvement of biostatisticians in clinical investigations discussed. |
| Senn and Julious  2009 | "In this paper, we seek to open up the debate on measurement, our objective is to encourage the medical statisticians to be more critical as regards this and to encourage them when undertaking collaborative research to contribute actively to the issue of measurement". | Statisticians on measuring in clinical trials | Text and opinion | None reported. | Role of medical statisticians in planning and analysing trials and in developing improved methods. Decisions taken by both physician and statistician described. |
| Sherrill et al.  2009 | “The authors summarize points for consideration generated in a National Institute of Mental Health (NIMH) workshop convened to provide an opportunity for reviewers from  different disciplines—specifically clinical researchers and statisticians”. | Clinical researchers and statisticians in grant applications | Text and opinion | 1-day workshop in October 2004 on the statistics/data analysis section of application and the role of the statistician in the application’s development and in study phases. | Depth and quality of collaboration between statistician and clinical researchers in analysis section of application and in the aims, data collection plans and budget. Statistician should be qualified, trained and experienced. Role of mentorship described. |
| Siegel et al.  2004 | "In this paper, we explore the arguments for and against having the statistician who is responsible for the unblinded interim analyses also be (1) ‘independent’ in the sense that the statistician is uninvolved in future decisions about modifications to the ongoing trial, and (2) not be an employee of the sponsor." | Independent statisticians (DMC) | Text and opinion | None reported. | Roles of multiple statisticians throughout trial described. Importance of independence of statistician described. |
| Sismondo  2009 | “This paper reports on a conference of an international association of publication planners. It describes and analyses their work in an ecological framework that relates it to marketing departments of pharmaceutical companies, medical journals and publishers, academic authors, and potential” | Publication planning in the medical sciences | Text and opinion | None reported. | Discussion on the role of publication planners of pharmaceutical company sponsored research. Data is analysed by statisticians but their work is rarely acknowledged. |
| Snapinn et al.  2004 | “The role of the unblinded sponsor  statistician” | Unblinded sponsor statisticians  (DMC) | Text and opinion | None reported. | Unblinded statistician is typically an employee of the sponsor. Advantages are discussed including: statistician being knowledgeable of treatment, background and objectives, ensuring quality of the analysis conforms to rigorous standards and confidentiality is maintained. |
| Snow et al.  2015 | “Herein, we describe the Hepatitis C Antiviral Long-Term Treatment against Cirrhosis (HALT-C) trial publication processes, which may prove a useful model for scientists and administrators  faced with data collection, analysis, and manuscript prioritization decisions in other complex studies with large databases of interrelated data on one population of study patients.” | Clinical researchers publishing in clinical trials | Empirical | ‘The HALT-C trial included a data coordinating centre (DCC) and clinical centres that recruited and followed more than 1,000 patients. Publication guidelines were approved by the steering and publications committee monitored publication process from selection of topics to publication’. | Publication guidelines are described and be implemented early in a trial. Role and collaboration of investigators and statisticians described. |
| Sosa et al.  2009 | “In this study, we measured the quality of peer-reviewed surgery manuscripts by using novel instruments to measure clinical, methodological, and overall quality of the reports in a double  blinded, randomized fashion.” | Evaluating surgery literature | Review | 120 clinical surgery manuscripts were selected from 1998 in 5 peer-reviewed surgery and medical journals. | Quality of surgery manuscripts can be enhanced by including a statistician as a co-author for multi-institutional/interdisciplinary trials. |
| Thall  2002 | “In this paper I discuss various ethical issues that I have encountered while working as a biostatistician at M.D. Anderson Cancer Centre. I describe particular experiences and the ethical issues involved.” | Ethical issues in oncology biostatistics | Text and opinion | None reported. | Roles and responsibilities of statisticians in medical research described, in the context of ethical consequences and issues: decision-making, benefit-harm trade-offs, safety monitoring, adaptive randomisation, informed consent and publication bias. |
| Tidwell et al.  2019 | “The goals of this article are to provide an update to our prior findings, characterize the use of Bayesian methods in cancer clinical trials, and identify potential barriers and challenges for implementing these methods.” | Statisticians in Bayesian cancer trials | Empirical | Review of clinical trials submitted to the MD Anderson Cancer institutional protocol office between January 2009 and December 2013. 1,020 trials analysed with regard to Bayesian methods implemented for design or analyses for each trial. | Barriers to overcoming the implementation of Bayesian designs include: suitable software, increasing desire amongst biostatisticians and clinicians, extra effort to implement newer designs, improving the quality of reporting and supporting regulatory agencies. Statistician involvement, collaboration and benefits for career opportunities also described. |
| Todd et al.  2020 | “This Horizons review will consider the benefits, challenges and suggested solutions of harnessing routine data for the purposes of ageing research.” | Ageing research | Text and opinion | None reported. | Requirement of researchers to be trained in undertaking appropriate analysis of data . Collaborative exercises discussed from the design to interpretation of findings to help drive the outputs of big data research into routine practice. |
| Tsang  1998 | ‘The methods needed to extract solid and ethical evidence are usually only described in statistically-oriented textbooks that are often inaccessible and incomprehensible to many busy clinicians. This short article has been written with the aim of bridging this gap for general readers.’ | Clinical researchers | Text and opinion | None reported. | Importance of GCP described and a qualified, experienced biostatistician conducting data analysis. Improved availability of biostatisticians in Hong Kong. Collaboration with investigator described. Independent statistician also mentioned. |
| Tyson et al.  2016 | “To specify better stopping guidelines in the protocol for such trials, the clinical investigators and trial statistician should carefully consider the following kinds of questions” | Statisticians and clinical investigators – stopping guidelines in trials | Text and opinion | None reported. | Collaboration of both statistician and clinical investigator when preparing stopping guidelines. |
| Vail  1998 | "This paper presents a statisticians membership experience on the local REC (LREC) of a large teaching hospital, and seeks to encourage  more involvement of statisticians at this stage of the research process." | Statisticians in UK RECs | Text and opinion | None reported. | Few UK local RECs have access to a biostatistician. Roles, responsibilities, workload and issues encountered by biostatisticians on RECs are described including sample size review. This would require more voluntary involvement by statisticians. |
| Van Ness et al.  2010 | “The objective of this article is to identify a set of statistical challenges arising in research with older persons that should be considered conjointly in the practice of clinical research and addressed  systematically in the training of biostatisticians intending to work with gerontologists, geriatricians, and older study participants.” | Gerontology research | Text and opinion | None reported. | Discussion on resources, clarifying tasks, and facilitating training of researchers and allowing for the collaboration of biostatisticians with colleagues dedicated to the health conditions of older persons. Methodological contributions are described. |
| Welzing et al.  2007 | “This field report demonstrates the consequences and implications of the (2001/20/EC) directive for paediatric investigator-initiated trials.” | Paediatric IIT | Field report/ Text and opinion | None reported. | CRF should be designed in close cooperation between the database developer and the trial statistician. Statistician is responsible for the statistical planning, generation of the randomisation list and the statistical analysis. |
| Whately-Smith et al.  2014 | “This paper provides an introduction to utilities for statisticians working mainly in clinical research who have not had experience of health technology assessment work.” | Statisticians and utility values in health technology assessments | Text and opinion | None reported. | Skills of clinical statistician applied to ensure the robustness of trial design, data collection and analyses of data in utilities and health technology assessments. Proposals for collaboration are described. |
| Williamson et al.  2000 | “This paper discusses some of the issues surrounding statistical review by research ethics committees (RECs).” | Statisticians in RECs | Text and opinion | None reported. | Lack of statisticians on local RECs described. Role of statisticians play on RECs explained in the context of statistical issues that arise when reviewing REC applications of research studies. Qualifications and training of REC members also mentioned. |
| Wittes  2004 | "This brief paper describes methods by which the reporting statistician can gain the knowledge necessary to produce informative reports. It also emphasizes that reports produced for the purpose of making decisions during the course of a trial differ in purpose, and should therefore differ in form, from reports summarizing the data and results at the end of the trial.” | Statisticians in DMCs | Text and opinion | None reported. | Proposed FDA model for DMCs: separate reporting statistician (writing reports for DMC) from the coordinating centre statistician (responsible for the statistical trial design, day-to-day operations and final data analysis). Risk of inadequate reports being produced for DMC unless reporting statistician understands the trial and database. |
| Zelen  2006 | “This paper discusses some of challenges facing our profession if we are to continue to be relevant in the biomedical sciences” | Statisticians in biomedical science | Text and opinion | None reported. | Leadership roles in evidence-based policy, responsibilities, capabilities of statisticians described as well as collaboration. Shortage of expertise, demand increases and availability for training is diminishing. Importance of ICH E9 described. |

RECs: Research Ethics Committees; LREC: Local Research Ethics Committee; FDA: Food and Drug Administration; DMCs: Data Monitoring Committees; CRF: Case Report Form; IIT: Investigator Initiated Trials; BMJ: British Medical Journal; SAP: Statistical Analysis Plan; EMA: European Medicines Agency; National Institutes of Health (NIH); ICH: International Council on Harmonization; PI: Principal Investigator; AD: Adaptative Design; NEJM: New England Journal of Medicine; JAMA: Journal of the American Medical Association; CCTs: Controlled Clinical Trials; CTU: Clinical Trials Unit; TSC: Trial Steering Committee; MRC: Medical Research Council; HTA: Health Technology Assessment; IRB: Institutional Review Board; EFSPI: European Federation of Statisticians in the Pharmaceutical Industry; PSI: Statisticians in the Pharmaceutical Industry; ADAPT-IT: Adaptive Designs Advancing Promising Treatments into Trials; ACTs: Adaptative Clinical Trials; TM: Translational Medicine; ABPI: Association of the British Pharmaceutical Industry; RCTs: Randomised Controlled Trials; HALT-C: Hepatitis C Antiviral Long-Term Treatment against Cirrhosis;
