# Supplementary material for: Factors influencing the statistical planning, design, conduct, analysis and reporting of trials in health care: A systematic review
Source: Contemp Clin Trials Commun. 2022 Jan 29;26:100897. doi: 10.1016/j.conctc.2022.100897 (PMC8842005; doi:10.1016/j.conctc.2022.100897)
Supplement: Multimedia component 3 [file mmc3.docx]

**Appendix C: Supplementary text**

*Funding and budgeting for statistician’s time*

Although it is difficult or impossible to perform an investigator-initiated trial without the cooperation of multi-disciplinary team members, including statisticians and data managers, this undoubtedly increases the expenses considerably (Welzing et al., 2007). The concern of insufficient funds for statisticians was described (Adams-Huet and Ahn, 2009; James 1980) and specifically in the context of DMCs (Bryant, 2004), the design of adaptive trials (Dimairo, Julious et al., 2015), stifling the creation of novel techniques in statistical theory and trial methodology (James, 1980). In the US, however, National Institutes for Health (NIH) grant applications for large RCTs require the involvement of a biostatistician prior to receiving funding for a study, for tasks including: justifying sample sizes (Li et al., 2018). The salaries of statisticians were questioned. Altman Goodman and Schroter (2002) reported that only 37% of biostatisticians reported having been paid for their work on a clinical research project (46% of these being RCTs).

*Ethical duties of statisticians*  

The relationship between statistics and ethics, and ethics committees emerged as another key factor. The routine professional activities of biostatisticians in health research are likely to have ethical consequences (Atici and Erdemir., 2007). For this reason, a number of authors advocated for biostatisticians to sit on RECs (Williamson et al., 2000; Atici and Erdemir., 2007; Vail, 1998). There was congruence in the literature, as the membership of statisticians on RECs in the UK and IRBs in the USA were reported as being a necessity, yet were not mandatory (Vail, 1998; Lin and Lu, 2014). However, it was noted that RECs, or institutional review boards (IRBs), often lacked both statistical and data management support to appropriately assess the safety risks of trials (Vail, 1998; Williamson et al., 2000; Lin and Lu, 2014) and lacked a fundamental understanding of statistics and study design (Vail, 1998). Some authors also noted the shortage of statisticians and the associated workload may limit on their availability to contribute to RECs (Vail, 1998; Williamson et al., 2000). The statistical queries of therapeutic intervention studies in REC applications are described (Vail, 1998; Williamson, 2000) and include: blinding, randomisation, placebo, groups, clarity/crossover, benefit from participation, information, effect size and sample size) and how statisticians on local RECs balance their input between the scientific assessment and lay concerns.

It is “*ethically imperative*” that statisticians conduct the best and most appropriate design and data analysis possible (Thall, 2002; Atici and Erdemir, 2007). Some authors (Adams-Huet and Ahn, 2009; Sherrill et al., 2009; Li et al., 2018) described the importance of the American Statistical Association (ASA) guidelines in the context of collaboration between clinical researchers and biostatisticians and making statistical decisions ethically. While not specific to clinical trials, the ASA guidelines (2018) for statistical practice highlights the professional integrity (also described by Sato and Yoshimura (1998) in the International Statistical Institute's Declaration of Professional Ethics) and accountability of a statistician when it comes to working with data, as well as their input in the methodology of a study and discusses the need for statisticians to work in a transparent manner (Ethical Guidelines for Statistical Practice, 2018).

*Multifaceted roles of the statistician in trial design*

The role of the statistician in the experimental design of a trial has been reported as a multifaceted one (Breslow, 1978), varies widely and includes: defining hypotheses, protocol writing, reading the literature and finding gaps in the knowledge, calculating power and sample sizes, of identifying sources of variation, determining endpoints or outcomes, specifying eligibility criteria, creating randomisation procedures, in data simulations and benchmarking studies (Breslow., 1978; James, 1980; Tsang, 1998; Grobler et al., 2001; Grieve, 2002; Adams-Huet and Ahn, 2009; Sherrill et al., 2009; Matcham et al., 2010; Phillips et al., 2013; Harman et al., 2015; Snow et al., 2015; Guetterman et al 2015; Califf, 2016; Boulesteix, Wilson and Hapfelmeier, 2017; Gamble et al., 2017; Li et al., 2018; Meeks et al., 2018; Papageorgiou et al., 2019). Four authors (Breslow, 1978; Thall 2002; Bierer et al., 2016 Tyson et al., 2016) described the necessary role of the statisticians in defining safe and formal early stopping rules for trials in collaboration with clinicians (Tyson et al., 2016).  The FDA 1988 Guideline ('Format and content of the clinical and statistical sections of an application') concludes that the onus of the statistical section of an application falls on the "*responsible statistician*". This guideline provides detailed information on the required statistical content of an FDA application being submitted to the FDA. While roles are not described explicitly, the guideline emphasises that the sponsor should consult a biostatistical reviewer and statisticians collaborating with sponsors is described (Breslow, 1978; Tsang, 1998; Adams-Huet and Ahn, 2009).

Communication can only be improved if both the clinician and the statistician can fully appreciate the other's perspective (Breslow, 1978; Bradstreet, 1992), or that between methodologists (including statisticians) and other health scientists (Ioannidis et al 2014). Neither statisticians nor PIs can conduct their tasks and make key decisions alone, but require the input of a team with the appropriate skills, to improve the methodology of the study (Crewson and Applegate, 2001; Perneger et al., 2004; Lewis, 2008; Califf, 2016). This encourages an environment of "*peer support*" (Todd et al., 2020), where team members are provided with statistical guidance yet make core decisions themselves (Grieve, 2002).

Investigators should be engaged as early as possible in the design stage of a trial to ensure the most critical research question(s) are defined, the data collected can be used to answer the research question(s) and that the results can provide a basis for additional studies (Meeks et al 2018). The early involvement of statisticians and investigators in creating Case Report Forms (CRFs) was described by seven authors (Crewson and Applegate, 2001; Grobler et al., 2001; Grieve, 2002; Welzing et al., 2007; Hattemer-Apostel, 2008; Adams-Huet and Ahn, 2009; Li et al., 2018), including their interaction with database developers and data management teams (Welzing et al., 2007; Hattemer-Apostel, 2008). , including the design of electronic CRFs and a specialised data management system (Grobler et al., 2001). Further detail is provided regarding the importance of this interaction between a database developer, the data management team and the trial statistician, in assuring valid documentation of data in the database and appropriate statistical evaluation (Welzing et al., 2007; Hattemer-Apostel, 2008), the collection of an appropriate quantity of data (Grobler et al, 2001; Crewson and Applegate, 2001; Hattemer-Apostel, 2008) and the clean-up of data (Califf, 2016).

A recommendation from the literature is that statisticians should avoid the use of "*loose language*" (Carroll, 2009), as "*technical jargon”* can create the perception that only statisticians are able to fully appreciate detailed statistical questions (Grieve, 2002). Statistical terminology however, is not meant to impede the work of young investigators, but rather facilitate and improve their interaction with a statistician (Bradstreet, 1992), as "*a valuable resource*" (Adams-Huet and Ahn., 2009) and create a nuanced language (Altman, Goodman and Schroter., 2002).

Multiple statisticians with different areas of expertise may be required in a research study (Adams-Huet and Ahn., 2009) but may make differing recommendations for similar situations (Ellenberg, 1990). Disagreements between statisticians therefore do arise, including the reluctance to try novel statistical methods (Grieve, 2002; Senn and Julious, 2009; Dimairo, Boote et al., 2015). Such claims of sticking to traditional methods should not be accepted as they are "*unscientific*" (Senn and Julious, 2009) and may be the result of a 'generation effect' - where some senior members of the statistical community may be reluctant to try novel adaptive designs and abandon *'standard'* practices (Dimairo, Boote et al., 2015).

*Analysis*

Discussion on the analysis of a trial is confined to roles and responsibilities of statisticians and those with statistical expertise. The role of statisticians, or "*statistical consultants*", is to help analyse data so that results can help to answer the research question (Boulesteix, Wilson and Hapfelmeier., 2017). The goal, however, of applied statistical researchers (Boulesteix, Wilson and Hapfelmeier., 2017) is to create novel analysis methods and tools (Senn and Julious, 2009). Snow et al (2015) discussed the importance of a second statistician performing quality control review analyses, with the potential for re-analyses if needed. The role of the statistician also extends to planning for an interim analyses in collaboration with the PI (Crewson and Applegate, 2001), in order to minimise the chance of bias (Lin and Lu, 2014).

*Role of the statistician in the preparation of the Statistical Analysis Plans*

A study by Gamble et al (2017) proposed 55 items to assist statisticians and other researchers when creating statistical analysis plans (SAPs) for trials. Several benefits to having clear and comprehensive SAPs were identified by the authors, including supporting the reproducibility of trials, improving the integrity of trial conduct and reporting, and reducing bias. Authors emphasised the importance of pre-specifying the SAP (Pyke et al., 2010), statisticians performing analyses according to their SAP (Snow et al., 2015), and documenting any data manipulations (Manamley et al., 2016). This was also described in the context of falsified data and the potential for fraud in trials (Pyke et al., 2010). Efforts to reduce analysing the data in a biased manner are also described in the context of blinding both the statistician and the investigator (Tsang, 1998).

*Reporting*

Relevant discussion on reporting in trials largely revolved around the role and work of statisticians. These mostly centre around the statistician's responsibility to honestly report studies, especially negative studies (Papageorgiou et al., 2019), using clear and accurate language that reflects the statistical analyses in an ethical manner (Thall, 2002). Discussion on the disadvantaged authorship of statisticians and conflicts of interests were also common.

A number of authors (Altman, Goodman and Schroter., 2002; Scales et al., 2005; Koletsi et al., 2012; Cullati et al., 2016) discussed the impact of not having a statistician on their research study. The involvement of statisticians were strong predictors for study publications being correctly labelled as RCTs (Koletsi et al., 2012), and had fewer “*scientific difficulties*” in the design, data management and analysis and publication writing (Cullati et al., 2016). On the other hand, papers with no statisticians or epidemiologists were more likely to be rejected by journals, before being sent for peer review (Altman, Goodman and Schroter., 2002) or have statistical inaccuracies in the design and analysis sections of publications (Scales et al., 2005, Ioannidis et al., 2014). The involvement of statisticians in CONSORT working groups to create and publish recommendations for reporting trials have been revised and extended (CONSORT Statement, 2010; Matcham *et al*., 2010).

*Engaging methodologists in trial interpretations and encouraging transparent reporting*

Methodologists (including epidemiologists and biostatisticians) are reported to play an important role in the methods section of trial publications (Delgado-Rodriguez et al., 2001). Several authors quantified the involvement of epidemiologists and biostatisticians in publications and found they improved the quality of the publication (Sosa et al., 2009; Delgado-Rodriguez et al., 2001; Kloukos et al., 2015; Papageorgiou et al., 2019).

Munro (1993) refers to the Declaration of Helsinki, where the physician is mandated to "*preserve the accuracy of results*" when publishing. Several authors recognise the responsibilities of investigators in the reporting of trials articles (Califf, 2004; Matcham et al., 2010; Pallman et al., 2018). This includes having the final responsibility of interpreting results of a study accurately, fairly, honestly and in a "*logical and scientific manner*" (Tsang, 1998), "*free of bias"* (Munro, 1993), while being aware of the potential drawbacks in the interpretation and reporting (Pyke et al., 2010; Pallman et al., 2018).

The role of a statistician has been described as a ‘multifaceted’ one (Breslow, 1978), where the FDA Guideline (1988) describes the onus of statistical sections of trial applications falling on the ‘responsible statistician’. Statisticians play a key role as either “statistical consultants” (Boulesteix, Wilson and Hapfelmeier., 2017) in analysing study data or as applied statistical researchers to create novel analysis methods and tools (Senn and Julious, 2009).

*Lack of acknowledgement of statisticians: authorship*

The International Committee of Medical Journal Editors (ICMJE)’s guidance regarding authorship, published in 2005 was cited by two authors (Sismondo, 2009; Matcham et al., 2010). One stated that "*authorship credit should be based on substantial contributions to conception and design, or acquisition of data, or analysis and interpretation of data*" (Sismondo, 2009) and the other that "*the person responsible for statistical aspects of the trial should be recognised as an author*" (Matcham et al., 2010).

*Conflicts of interest*

Conflicts of interests (COIs) are another factor that can negatively influence the reporting of trials and the interpretation of results. Munro noted that the Royal College of Physicians refer to the responsibility of the investigator in ensuring there is prior agreement with the sponsor of the study that the study results can be submitted to journals chosen by the investigator and that the sponsor will not influence the findings (Munro, 1993). Neither the investigator nor co-authors should be pressured by a sponsor company to withhold the publication of trial results or report findings they feel are inappropriate (Matcham et al 2010). While almost all articles that discussed reporting in this review referred to the necessary role of statisticians, Sismondo (2009) asserts their experiences of sponsored trials being handled by Contract Research Organisations (CROs), where the company statisticians conduct the analysis but the papers are written by professional medical writers and a publication planning team, while also maintaining control of the data (Sismondo, 2009). Similarly, Munro (1993) discusses how publications, may be at the discretion of the sponsoring pharmaceutical company.

Bryant (2004) and Ellenberg and George (2004) note that it is rare that statisticians would intentionally disclose interim results in an attempt to bias or influence the study or its findings and are usually free of financial COIs, and are reasonably independent of trial leadership, unlike investigators or sponsors (Ellenberg and George, 2004). However, statisticians are perceived to have a COI due to their abundant knowledge and experience in the area of research and therefore an increasing likelihood they have a connection with a study in that area (Ellenberg, 2012). COIs and financial interests in the sponsor or a company, if any, of statisticians should be declared (Matcham et al 2010). On the other hand, Ioannidis et al. (2014) are of the opinion that the influence of "*conflicted stakeholders*" in these phases of trials should be reduced considerably and instead involve stakeholders without financial COIs.

*Increasing the statistical knowledge of non-statisticians*

Two authors (Breslow, 1978; Ellenberg, 1990) raise concerns about non-statisticians conducting several statistical tasks and "*manipulating data files*" (Breslow, 1978) using various software, without the support of a professionally trained statistician. While it was not found to be essential for clinicians and investigators to have in-depth statistical knowledge, a number of authors (Tsang, 1998; Perneger et al., 2004; Adams-Huet and Ahn, 2009; Califf, 2016; Pallman et al., 2018) indicated that they should have fundamental knowledge and a conceptual understanding of methods and statistical reasoning, to ensure their interaction with biostatisticians (and epidemiologists) are as productive as possible (Perneger et al., 2004). Several authors advocated for training in biostatistics and utilising data (Crowley et al., 2018; Tidwell et al., 2019; Todd et al., 2020), through training fellowships (Dimairo, Boote et al., 2015) (specifically funded by industry which will benefit companies in the long term (Zelen, 2006)), and education of the wider research community in adaptive designs (Coffey et al., 2012), as well as in statistical reporting (Prescott et al., 1999). The level of training in biostatistics and quantitative methods received by investigators was reported (Cullati et al., 2016; Crowley et al., 2018). Bradstreet (1992) and Dhar and Kianifard (2006) describe courses they conducted for non-statisticians in pharmaceutical companies while Ellenberg (1990) discussed the use of more pedagogical techniques when teaching. The benefits of these courses are reported and include: improving the communication between clinical researchers and statisticians, allowing the statisticians to gain teaching experience and increasing the recognition of statistical contribution to a research study (Bradstreet, 1992; Dhar and Kianifard, 2006).
